# *FLA1*, Enhancing GA_3_ Contents in Flag Leaf Lamina Joint, Increases Flag Leaf Angle to Improve Outcross Rate and Hybrid Rice Seed Production

**DOI:** 10.3390/plants15030446

**Published:** 2026-01-31

**Authors:** Zhiyao Dong, Dalu Li, Xiaoxiao Hu, Xuanchi Liu, Nuoya Fei, Guocan Wu, Erbao Liu, Xiaojing Dang, Siyuan Zeng, Yuzhu Chen, Delin Hong

**Affiliations:** 1 Jilin Provincial Key Laboratory of Plant Resource Science and Green Production, Jilin Normal University, Siping 136000, China; dzy0903@jlnu.edu.cn (Z.D.);; 2 State Key Laboratory of Crop Genetics and Germplasm Enhancement, Nanjing Agricultural University, Nanjing 210095, China; 3 Ningde Institute of Agricultural Sciences, Ningde City 355017, China; 4 WisDelivery Biotechnology (Wuhan) Co., Ltd., Wuhan 430075, China

**Keywords:** rice, flag leaf angle, *FLA1*, favorable alleles, hybrid seed production

## Abstract

Flag leaf angle (FLA) in rice (*Oryza sativa* L.) is one of the important traits affecting F_1_ seed production by mechanization. Here, we report the map-based cloning and functional characterization of the *FLA1* (*FLAG-LEAF-ANGLE 1*) gene, which resides at a major-effect quantitative trait locus (QTL). Through cell morphological observations and exogenous hormone treatment assays, we demonstrate that gibberellin (GA) modulates rice FLA by altering both the number of cell layers and cell length. Combining genetic and molecular biological analyses with genetic complementation and gene overexpression assays, we elucidated and validated the biological function of *FLA1*. In addition, we found that *FLA1* is constitutively expressed and encodes a protein localized to both the cell membrane and nucleus. Via RT-qPCR assays, we further demonstrated that the *FLA1^fla-R^* allele from the rice accession fla-R enhances GA biosynthesis by upregulating the expression of *CLA1* and *GA20ox2*. Furthermore, yeast two-hybrid assays revealed that auxin-repressed protein 1 (ARP1) interacts with FLA1, suggesting a potential role of this interaction in the modulation of rice FLA. Collectively, our results demonstrate that optimizing rice FLA via molecular manipulation of *FLA1* can resolve the problem of flag leaf shearing during F_1_ hybrid rice seed production without compromising F_1_ hybrid seed yield, thereby facilitating mechanized F_1_ hybrid rice seed production.

## 1. Introduction

**Figure 1:**
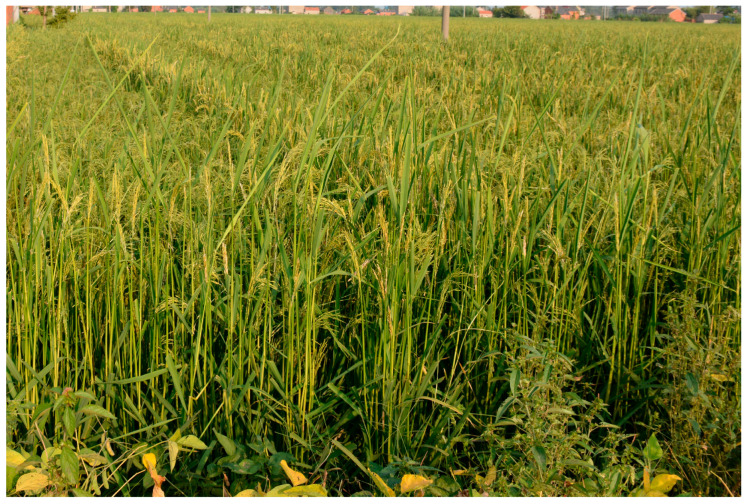
Hybrid rice F_1_ seed production field at the grain filling stage in Jiangsu Province, China.

Rice *(Oryza sativa* L.) serves as the staple food for over 3 billion people worldwide, with a global annual planting area of approximately 160 million hectares spanning 117 countries and an average yield of 4.4 t ha^−1^ [1]. With the growing global population and declining arable land, improving unit yield is imperative to ensure global food security [2,3,4]. Thus, boosting the rice yield is a critical priority for agricultural development and livelihood improvement worldwide [5]. China’s 48-year commercial cultivation of hybrid rice has confirmed that heterosis utilization is an effective strategy to increase rice unit yield. Field practices show that the small flag leaf angle (FLA) of the male parent in hybrid rice seed production often impairs the pollination efficiency of sterile lines [6,7]. To remove cross-pollination barriers, farmers typically manually cut off one-third to half of the parental flag leaf blades at the initial heading stage (Figure 1). This practice is not only labor-intensive but also requires high operational skills to avoid damaging young panicles. Therefore, developing male sterile lines with a large flag leaf angle is conducive to the flag leaf cutting step and facilitates the mechanization process of hybrid rice seed production.

Rice leaf angle, defined as the angle between the main stem and leaves, is a key indicator of rice plant type. Its regulatory mechanisms are mainly categorized into two types: hormone synthesis and signal transduction pathways and non-hormonal regulatory mechanisms. Detailed research findings are as follows: (1) defects in key brassinosteroid (BR) synthesis genes reduce leaf angle. For example, *dwarf4-1* [8], *brd1* [9], *d2* [10], and *d11-1* [11] all showed an erect plant type. (2) Blocked BR signaling decreases leaf angle: for instance, U-type cyclin (CYCU4;1), regulated by BR signal transduction in *d2-2* and *d61-1*, inhibits the proliferation of specific cells on the abaxial surface of leaf lamina joints, thereby reducing leaf angle [12]. Additionally, the bHLH transcription factor *OsbHLH98* modulates BR signaling to suppress the negative regulatory effect of *OsBUL1* on leaf angle [13]. (3) BR signal transduction via key gene expression induces leaf angle inclination: overexpression of BR signaling key genes (*DLT*, *GSK2*, *BU1*, and *ILI1*) increases leaf angle [14,15,16,17,18]. In addition, the *OsFLP* functions with *OsGSK1* and *OsBZR1* in BR signaling to maintain optimal leaf angle by modulating the lignin deposition in mechanical tissues of the lamina joint [19]. Moreover, *LPA1* in mutant *lpa1* controls leaf lamina joint curvature by inhibiting auxin signaling associated with C-22 hydroxylation and 6-deoxy-brassinolide, leading to leaf angle inclination [20]. (4) Auxin (IAA) and gibberellic acid (GA) positively regulate rice leaf angle enlargement: for example, auxin response factor *OsARF19* in mutant *osarf19* promotes leaf angle enlargement by positively regulating *OsGH3-5* and *OsBRI1* [21]. In addition, the *LC2* (leaf inclination2) encodes a vernalization insensitive3-like protein regulated by gibberellin, which is concentrated in the rice leaf lamina joints, making the leaf angle of mutant *lc2* larger [22]. (5) SiRNA interference reduces leaf angle; for example, *OsDCL3* produces 24-nt siRNAs, which induce leaf angle inclination [23]. A summary of these studies reveals four cytological features affecting rice leaf angle changes: (1) altered longitudinal cell length near leaf lamina joints [24]; (2) the second is that the number of cells near the leaf lamina joints has changed [12,22]; (3) the third reason is the strength of the mechanical tissue at the leaf lamina joints has changed [19,25,26]. (4) The last point is transcription factor *OsWRKY72* controls rice leaf angle by regulating *LAZY1*-mediated shoot gravitropism [27]. These cytological changes are coordinately regulated by multiple hormones and genes, and the underlying regulatory mechanisms remain complex and require further exploration.

The rice flag leaf angle (FLA) is defined as the angle between the main stem and flag leaf blade [28]. Previous studies reported that rice FLA is controlled by one pair of major genes and multiple minor gene pairs, with the small-angle phenotype showing partial dominance. To date, a total of 71 FLA-related QTLs have been characterized, which are widely distributed across all 12 rice chromosomes, with distinct patterns in their genetic effects and validation frequencies reported by different research groups [6,7,29,30,31,32,33,34,35,36,37,38,39,40]. Chromosome 1 harbors 10 FLA-related QTLs, with *qFLA1* (linked to marker RM6696). Specifically, chromosome 1 houses 10 QTLs, among which *qFLA1* (linked to marker RM6696) has been repeatedly detected by three independent teams, with its phenotypic variation explained (PVE) values ranging from 5.4% to 12.5%; chromosome 2 contains 6 QTLs with PVEs spanning 5.4–13.8%; and chromosome 3 carries 6 QTLs, including *qFLA3* (RM5639-RM232) validated by two research groups, with corresponding PVEs varying from 4.74% to 12.97%. Chromosome 4 harbors only 4 QTLs, showing relatively modest contribution rates of 4.4–6.1%, whereas chromosome 5 encompasses 5 QTLs that exhibit the broadest PVE range (5.8–37.71%) among all chromosomes. Seven QTLs map to chromosome 6, one of which—*qFLA6* (RM314-RM136)—has been confirmed by two separate studies, with PVEs falling between 4.2% and 12%. Notably, several chromosomes carry QTLs with remarkably high genetic effects: chromosome 7 carries 5 QTLs with PVEs of 6.6–48.26%; chromosome 8 features 8 QTLs, among which *qFLA8* (RM3572-RM5556) and *qFLA8-1* (RM8265-RM6215) have each been repeatedly identified, and their contribution rates span 5.9–48.24%; chromosome 9 contains 4 QTLs, with *qFLA9* (RM3909) validated by two teams and PVEs ranging from 5.56% to 48.29%. In contrast, chromosome 10 has the fewest QTLs (only 2) with contribution rates of 5.19–16.28%; chromosome 11 harbors 9 QTLs characterized by the lowest minimum PVE (1.21%) and a maximum of 16.24%; and chromosome 12 closes the spectrum with 5 QTLs whose PVEs range from 7.74% to 28.75%. Collectively, these findings highlight the extensive and complex genetic basis governing rice FLA variation, and the identification of stably inherited, major-effect QTLs lays a critical foundation for subsequent gene cloning and molecular breeding of ideal plant architecture in rice. Despite these advances in the characterization of FLA-related QTLs, in-depth investigations into the underlying regulatory mechanisms of FLA development remain limited. For example, Dong et al. (2018) used genome-wide association analysis to show that bHLH16 subfamily members modulate FLA and act as brassinosteroid (BR) and indole-3-acetic acid (IAA) response factors [41]. Huang et al. reported that two auxin response factors, *OsARF6* and *OsARF17* (highly expressed in lamina joints), regulate FLA in response to auxin [42]. In hybrid rice seed production, gibberellin (GA) spraying during panicle differentiation is commonly used to increase FLA and improve seed production efficiency. Gibberellin A_3_ (GA_3_), a key endogenous rice hormone, promotes cell elongation and enlarges FLA [43]. However, its regulatory mechanism remains unclear. Collectively, the molecular mechanisms governing rice FLA are not fully elucidated and require further exploration.

The objectives of this study were to (1) clone the favorable allele of *FLA1* (*FLAG-LEAF-ANGLE 1*) from accession fla-R, which is located at a major-effect QTL regulating rice FLA; (2) elucidate the regulatory mechanism of *FLA1* in controlling flag leaf angle; and (3) determine the role of *FLA1* in improving the outcrossing rate.

## 2. Results

### 2.1. FLA1 Is a Major Locus Controlling the FLA of Rice

**Figure 2:**
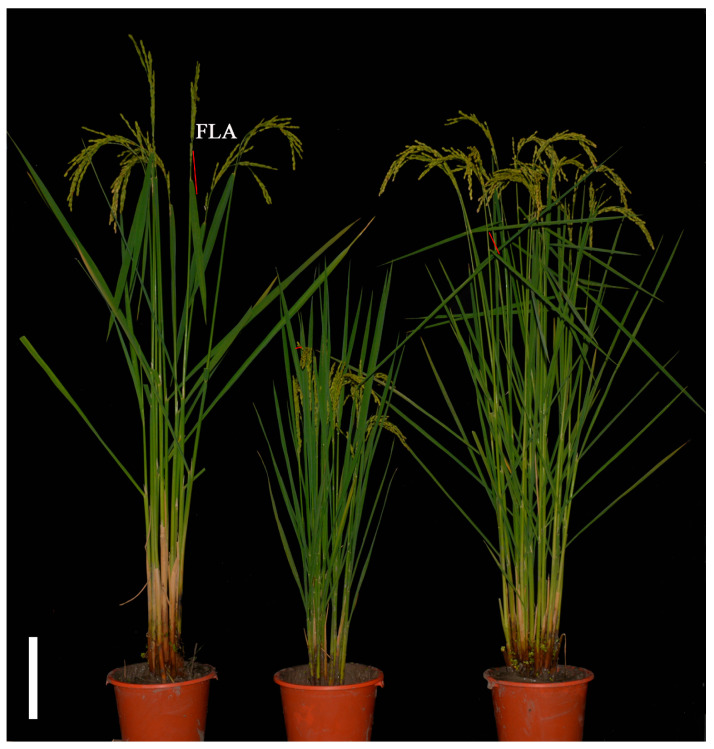
Plant morphology of A7444 (left), 863B (middle) and fla-R (right) at filling stages. (bar = 10 cm).

A BC_1_F_1_ population derived from a cross between 863B and A7444 was constructed for SSR marker-based genetic map construction. 863B is the maintainer line of a released *japonica* rice hybrid combination in Jiangsu Province, China, with a flag leaf angle (FLA) of 15–25° (Figure 2).

**Figure 3:**
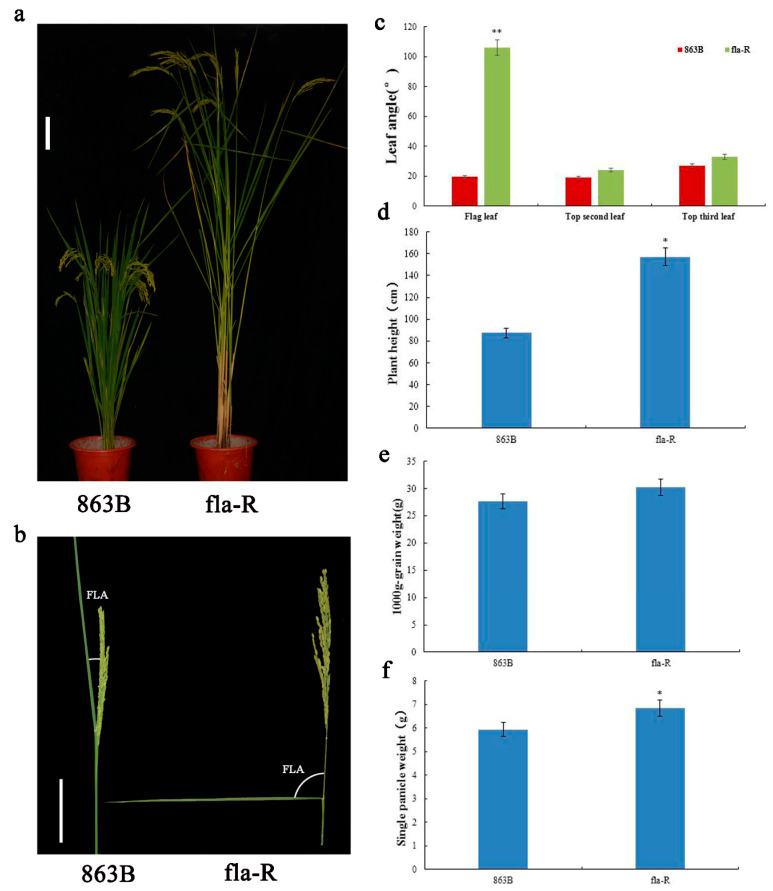
Phenotype of 863B and fla-R at the grain filling stage. (**a**) Whole plant phenotypes of 863B and fla-R at the grain filling stage (bar = 10 cm); (**b**) morphology of panicles and flag leaves of 863B and fla-R, showing flag leaf angle (FLA) (bar = 2.5 cm); (**c**) comparison of leaf angle (flag leaf, top second leaf, top third leaf) between 863B and fla-R; (**d**) histograms of plant height comparison between 863B and fla-R; (**e**) comparison of 1000-grain weight between 863B and fla-R; (**f**) comparison of single panicle weight between 863B and fla-R. (**c**–**f**) taken the average of 10 samples; * and ** represent significant difference at *p* < 0.05 and *p* < 0.01 probability level by Student’s *t*-test (**c**), respectively.

A7444 is a glutinous *japonica* rice landrace from the Taihu Lake region of China, with an FLA of 120–160° (Figure 2). Linkage analysis of 87 SSR markers was performed to construct an SSR molecular marker linkage map (Supplementary Figure S2), with a total length of 1457.6 cM, 81 marker loci, and an average genetic distance of 17.9 cM. Two stable QTLs controlling FLA (Supplementary Table S1) were detected in the BC_1_F_1_ population, located on chromosomes 2 and 8, respectively. *qFla2* is flanked by SSR markers RM300 and RM145, while *qFla8* is between SSR markers RM6215 and RM8265; favorable alleles for both QTLs are derived from A7444. Using 863B as the recurrent parent and A7444 as the donor parent for SSR marker-based genotypic identification 192 individual plants were selected from the BC_3_F_3_ population developed. Subsequently, the obtained genotypic data were integrated with the corresponding FLA phenotypic data to construct a genetic map and perform QTL mapping analysis, thus further narrowing down the interval of the chromosomal segment harboring *qFla8*. This process revealed two linked loci (*qFla8-1* and *qFla8-2*) within the original *qFla8* segment (Supplementary Figure S3). *qFla8-1* is located between SSR markers RM6215 and RM3153, explaining 22.33% of the phenotypic variation with the favorable allele from 863B; *qFla8-2* is between SSR markers RM1309 and RM3491, accounting for 23.81% of the phenotypic variation with the favorable allele from A7444. Therefore, the BC_3_F_4_ population carrying the *qFla8-2* locus (FLA: 90–110°) was selected as the research object and named fla-R (Flag leaf angle recessive). As shown in Figure 3a–c, the FLA of fla-R was significantly larger than that of 863B (*p* < 0.01). No significant differences were observed between 863B and fla-R in the second and third leaf angles from the top, indicating that fla-R specifically exhibits flag leaf inclination with little effect on other leaf angles (Figure 3a,c). Additionally, plant height statistics showed that the average plant height of fla-R was 157.24 cm, while that of 863B was 87.52 cm (Figure 3d); this difference may be attributed to either incomplete homozygosity of the near-isogenic line (NIL) background or the pleiotropic effect of *FLA1*, as the latter’s potential role in regulating plant height has not been excluded. The average 1000-grain weight was 27.68 g for fla-R and 30.21 g for 863B, with no significant difference (Figure 3e). The single panicle weight of fla-R (6.84 g) was significantly higher than that of 863B (5.93 g) (*p* < 0.05) (Figure 3f).

### 2.2. GA Regulates FLA Rice by Affecting Changes in Cell Layer Number and Length

**Figure 4:**
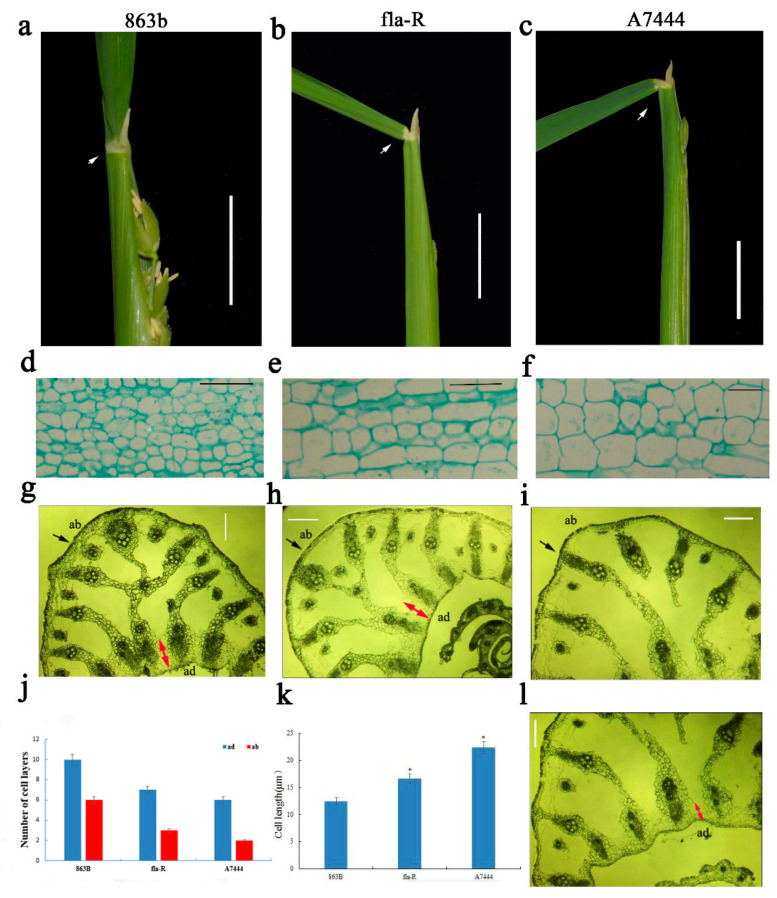
Morphological and anatomical differences of 863B (left), fla-R (middle), and A7444 at eighth stage of young panicle differentiation. (**a**–**c**) morphologies of flag leaf lamina joint in 863B, fla-R, and A7444. bar = 2.5 cm; (**d**–**f**) cell morphologies of longitudinal sections of proximal axial in 863B, fla-R, and A7444. bar = 50 μm. (**g**–**i**) cell morphologies of cross sections in 863B, fla-R, and A7444. bar = 150 μm; (**j**) comparison of the numbers of parenchymal cell layer at the abaxial (ab) and adaxial (ad) ends in the three genotypes. (**k**) Average cell length at the paraxial end in the three genotypes. (**l**) Cell morphology of adaxial of cross section in A7444. bar = 150 μm; * indicates significance at *p* < 0.05 probability level. White arrows indicate the flag leaf lamina joint; red arrows indicate the number of cell layers (ad).

To explore the cytological mechanisms underlying flag leaf inclination in fla-R, the flag leaf lamina joints of A7444, 863B, and fla-R were observed (Figure 4). In transverse sections sampled at the eighth young panicle differentiation stage, the number of adaxial (ad) cells in A7444 and fla-R was significantly lower than that in 863B (Figure 4g–j). In longitudinal sections, the length of abaxial (ab) longitudinal cells in 863B was significantly shorter than that in fla-R and also shorter than that in A7444 (*p* < 0.05) (Figure 4d–f,k). Additionally, histological observations at the sixth panicle differentiation stage revealed that the number of adaxial (ad) cell layers in A7444 and fla-R was significantly lower than that in 863B (*p* < 0.05) (Supplementary Figure S4). Therefore, the enlargement of FLA in fla-R may be attributed to two factors: (1) reduced number of adaxial cells; (2) elongation of abaxial longitudinal cells. Endogenous hormone determination in flag leaves and flag leaf lamina joints of 863B and fla-R at the sixth young panicle differentiation stage showed that the GA_3_ content in fla-R (0.8 ng/g) was significantly higher than that in 863B (0.09 ng/g) (*p* < 0.01), and the GA_3_ content in fla-R was also higher than its IAA (0.18 ng/g) and BR (0.06 ng/g) contents (*p* < 0.05) (Supplementary Table S2).

**Figure 5:**
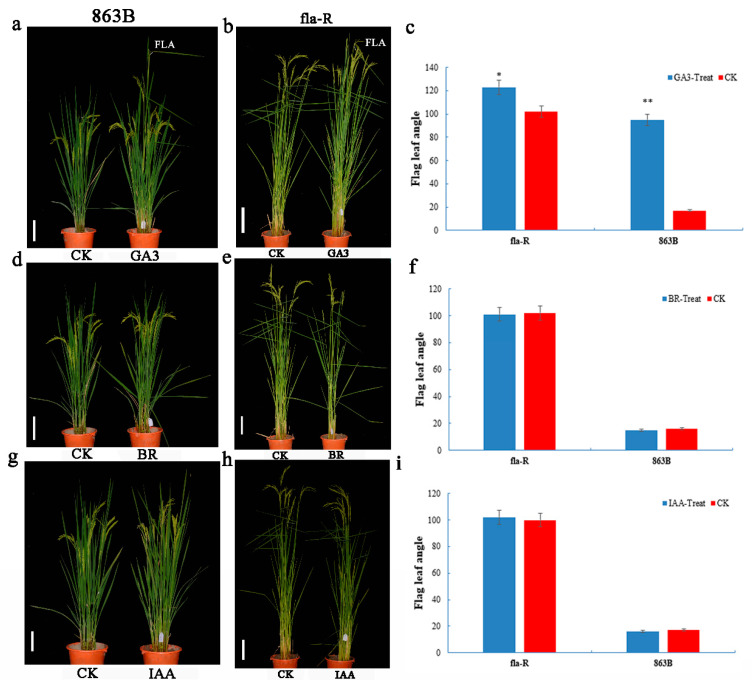
Changes of flag leaf angle 7 days after GA_3_, BR, and IAA treatments. (**a**) Comparison of the flag leaf angles in 863B between water (CK) and GA_3_ (10 μM) treatment. bar = 10 cm; (**b**) comparison of the flag leaf angles in fla-R water (CK) and GA3 (10 μM) treatment. bar = 10 cm; (**c**) histograms of the flag leaf angles after GA_3_ treatment and CK in 863B and fla-R; (**d**) comparison of the flag leaf angles in 863B between water (CK) and BR (1 μM). bar = 10 cm; (**e**): comparison of flag leaf angles in fla-R between water (CK) and BR (1 μM). bar = 10 cm; (**f**) histograms of the flag leaf angles after BR treatment and CK in 863B and fla-R; (**g**) comparison of flag leaf angles in 863B between water (CK) and IAA (20 μM). bar = 10 cm; (**h**) comparisons of the flag leaf angle in fla-R between water (CK) and IAA (20 μM). bar = 10 cm; (**i**) histograms of the flag leaf angles after IAA treatment and CK in 863B and fla-R. * and ** represent statistically significant differences at the probability levels of *p* < 0.05 and *p* < 0.01, respectively.

These results indicate that at the sixth young panicle differentiation stage (the initial stage of FLA enlargement), GA_3_ synthesis in fla-R flag leaves (including flag leaf lamina joints) is at a high level with a strong synthesis signal. Hormone (IAA, BR, GA_3_) spraying experiments on fla-R and 863B showed that GA_3_ could increase the FLA of both fla-R and 863B (Figure 5a–c), while BR and IAA treatments did not cause significant changes in the FLA of fla-R or 863B (Figure 5d–i). Thus, GA_3_ may be the main factor regulating FLA enlargement in fla-R.

### 2.3. Map-Based Cloning of FLA1

**Figure 6:**
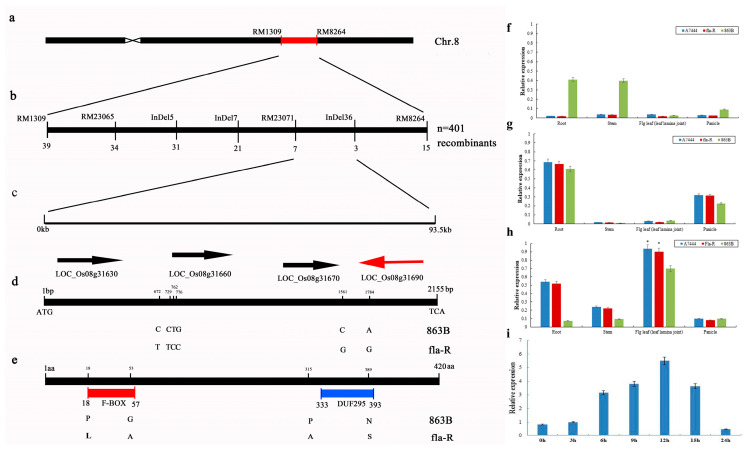
Map-based cloning and expression analysis of *FLA1.* (**a**) The *FLA1* locus was mapped to the marker region of RM1309-RM8264 on the long arm of chromosome 8 using the BIL population; (**b**) the *FLA1* locus was further delimited to a 93.5 Kb region between RM23071 and InDel36 using 401 recessive homozygous plants; (**c**) four open reading frames in the region of RM23071 and InDel36; (**d**) nucleotide sequence difference between 863B and fla-R at gene *FLA1*; the arrows indicate the positions of the four candidate genes; (**e**) amino acid sequence variation in the FLA1 protein between 863B and fla-R; (**f**) relative expression of *ORF1* (*LOC_Os08g3630*) in root, stem, flag leaf lamina joint, and panicle; (**g**) relative expression of *ORF2* (*LOC_Os08g3660*) in root, stem, flag leaf lamina joint, and panicle; (**h**) relative expression of *ORF4* (*LOC_Os08g3690*) in root, stem, flag leaf lamina joint, and panicle; (**i**) transient expression of *FLA1^fla-R^* after GA_3_ treatment in the young panicle differentiation stage. * represents significant differences at *p* < 0.05 probability level.

Genetic analysis of a BC_3_F_4_ population derived from selfing of BC_3_F_3_ (recurrent parent: 863B; donor parent: A7444), for which 163 individual plants were randomly selected for flag leaf angle (FLA) investigation, showed a 3:1 segregation ratio for the flag leaf angle (FLA) trait at the *FLA1* locus (119 plants with small FLA, 44 plants with large FLA; χ^2^ = 0.25 < χ^2^_0.05_ = 3.84), indicating that large FLA is recessive to small FLA. This Mendelian segregation pattern is jointly attributed to two key factors: first, three generations of backcrossing with 863B as the recurrent parent have eliminated most genomic segments (including minor QTLs for FLA) from the donor parent A7444, resulting in only the *FLA1* locus segregating in the BC_3_F_4_ population; second, *FLA*1 itself is a major-effect QTL with a strong genetic contribution, whose dominant-recessive inheritance dominates the phenotypic separation of FLA, and any residual effects of minor QTLs are masked by the prominent effect of *FLA1*. High-resolution mapping was performed using 401 recessive homozygous plants with large FLA (FLA > 85°) from a BC_3_F_4_ population of 4382 plants, delimiting the FLA1 locus to a 93.5-kb chromosomal region between the insertion/deletion (InDel) marker 36 and SSR marker RM23071 (Figure 6a–c). This region contains four annotated open reading frames (ORFs; National Center for Biotechnology Information: http://www.ncbi.nlm.nih.gov/, accessed on 11 October 2015): *ORF1* encodes a trehalose-phosphatase; *ORF2* contains a VQ domain; *ORF3* encodes a hexuronate transporter; and *ORF4* contains an OsFBX291 F-box domain and a DUF293 domain (Figure 6c, Supplementary Table S3). Sequencing of these four *ORFs* revealed no sequence differences in ORF3 between fla-R and 863B (Supplementary Figure S5a), while significant base sequence differences were observed in the other three *ORFs* (O*RF1*, *ORF2*, and *ORF4*). Thus, *ORF3* was first excluded as a candidate gene. Genome sequencing of *ORF4* (*LOC_Os08g31690*) showed six nucleotide mutations between fla-R and 863B (Figure 6d, Supplementary Figure S6), leading to four amino acid mutations: two in the F-box domain [Proline (863B, P)→Leucine (fla-R, L); Glycine (863B, G)→Alanine (fla-R, A)] and one in the DUF293 domain [Asparagine (863B, N)→Serine (fla-R, S)] (Figure 6e, Supplementary Figure S5b). Reverse transcription quantitative PCR (RT-qPCR) analysis showed that *ORF1* and *ORF2* were barely expressed in flag leaves and flag leaf lamina joints, whereas *ORF4* exhibited a relatively high expression level with a significant difference between fla-R and 863B (*p* < 0.05) (Figure 6f–h). fla-R plants at the sixth panicle differentiation stage were treated with GA_3_, and samples were collected at different time points for RT-qPCR. As shown in Figure 6i, transient expression of *ORF4* fluctuated significantly following exogenous GA_3_ application, with a peak at 12 h, indicating that *ORF4* may be regulated by GA_3_. Therefore, *ORF4* is the most likely candidate gene affecting FLA.

### 2.4. Confirmation of LOC_Os08g31690 as FLA1

**Figure 7:**
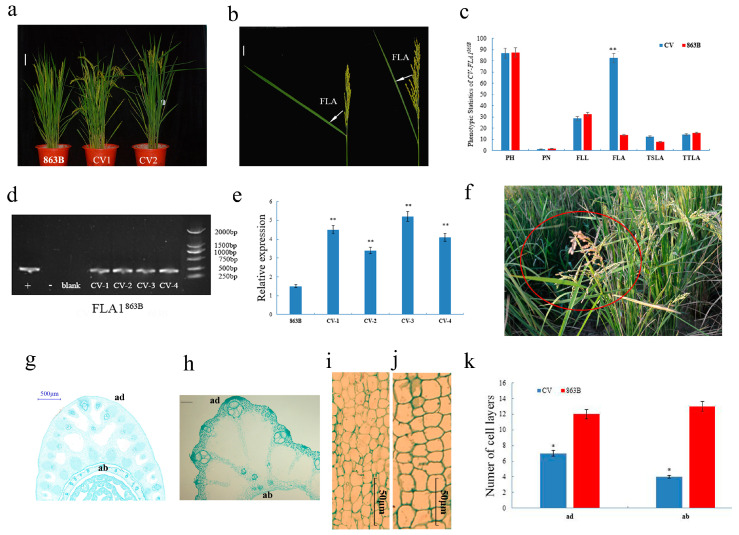
Phenotype of *FLA1^fla-^^R^* complementary lines. (**a**) Changes in FLA of Complementary Plants (bar = 10 cm); (**b**) comparison of flag leaf angle between transgenic leaf line CV1 (left) and 863B (right) (Bar = 2.5 cm); (**c**) comparison of agronomic traits (plant height, panicle number, flag leaf length, flag leaf angle, top second leaf angle, top third leaf angle) between transgenic complement lines and control 863B. (**d**) PCR amplification of the hygromycin resistance gene to confirm the positive transgenic plants, including positive control (+), negative control (−), blank control, and four independent complementary lines (CV1–CV4); (**e**) relative expression of *FLA1^flaR^* in 863B and four transgenic complement lines (CV1, CV2, CV3, and CV4); (**f**) Field performance of transgenic complementary lines, with the red circle indicating the target plants; (**g**) 863B flag leaf lamina joints transect (bar = 500 μm); (**h**) the *FLA1^fla-R^* complementary line flag leaf lamina joints transect (bar = 50 μm); (**i**) Longitudinal section of the flag leaf lamina joints in 863B (bar = 50 μm); (**j**) longitudinal section of flag leaf lamina joints in the transgenic complementary line (bar = 50 μm); (**k**) comparisons of cell layer numbers between 863B and the transgene complementary line. * represents significant differences at *p* < 0.05 probability level. ** represents significant differences at *p* < 0.01 probability level; ad = adaxial; ab = abaxial.

*FLA1^fla-R^* (*LOC_08g31690*, 2.1kb) was ligated into the expression vector *pCAMBIA1301*, transferred to 863B by the *Agrobacterium*-mediated method, and 12 T_3_ transgenic lines with large FLA phenotypse were obtained. (*CV-FLA1^863B^*, Figure 7). The primer *hyg*-F/*hyg*-R was used for PCR identification of *CV-FLA1^863B^*, and a 540 bp band was amplified in the transgenic positive line (Figure 7d). The expression level of *FLA1* in the transgenic complementary line was significantly increased (Figure 7e). The phenotypic statistical results of *CV-FLA1^863B^* showed that compared with 863B, only the FLA was significantly increased (*p* < 0.01) (Figure 7a–c,f). Microscopic observation of the flag leaf lamina joints demonstrates that the reduction in the number of cells at the distal end and the elongated longitudinal cells at the proximal end both control FLA changes (Figure 7g–k). In addition, *FLA1^fla-R^* was transferred into fla-R to obtain T_3_ transgene overexpression lines (*OE-FLA1^fla-R^*).

**Figure 8:**
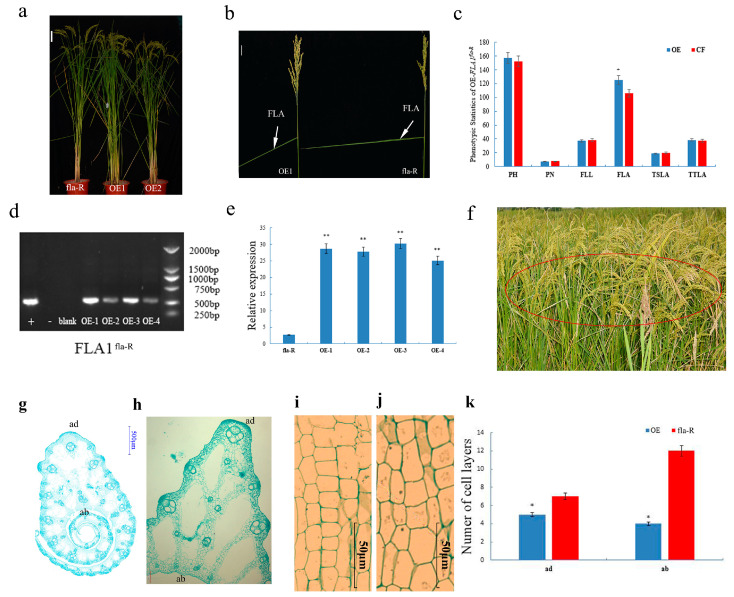
Phenotype of *FLA1^fla-R^* overexpression lines. (**a**) Whole-plant phenotypes of transgenic *FLA1^fla-R^* overexpression line (bar = 10 cm); (**b**) comparison of flag leaf angle between transgenic leaf line OE1 (right) and fla-R (left) (bar = 2.5 cm); (**c**) comparison of agronomic traits between transgenic overexpression lines and control fla-R; (**d**) PCR amplification of the hygromycin resistance gene to confirm positive transgenic plants, including positive control (+), negative control (−), blank control, and four independent overexpression lines (OE1–OE4); (**e**) relative expression of *FLA1^flaR^* in fla-R and four transgenic overexpression lines (OE1, OE2, OE3, and OE4); (**f**): field performance of transgenic overexpression strains, with the red circle indicating the target plants; (**g**) fla-R flag leaf lamina joints transect (bar = 500 μm); (**h**) transgene overexpression line flag leaf lamina joints transect (bar = 50 μm); (**i**) Longitudinal section of the flag leaf lamina joints in fla-R (bar = 50 μm); (**j**) longitudinal section of flag leaf lamina joints in the transgenic overexpression line (bar = 50 μm); (**k**) comparisons of cell layer numbers between fla-R and the transgene overexpression line. * represents significant differences at *p* < 0.05 probability level. ** represents significant differences at *p* < 0.01 probability level; ad = adaxial; ab = abaxial.

PCR identification of *OE-FLA1^fla-R^* amplified a 540 bp band (Figure 8d). As shown in Figure 8e, the expression level of *FLA1* in *OE-FLA1^fla-^*^R^ was significantly increased. The phenotypic statistics showed that the FLA of *OE-FLA1^fla-R^* increased significantly *(p* < 0.05) (Figure 8a–c,f). In addition, it can be seen from Figure 8c that there was no significant difference in other phenotypic traits. The observation results of *OE-FLA1^fla-R^* slices showed that the number of cells at the distal end of the flag leaf lamina joints was reduced, and the longitudinal cells at the proximal end were elongated (Figure 8g–k). The above experimental results (Figure 7 and Figure 8) show that the *FLA1* may be a functional gene affecting the change of FLA in rice.

### 2.5. FLA1 Exhibits a Relatively High Expression Level in the Flag Leaf

**Figure 9:**
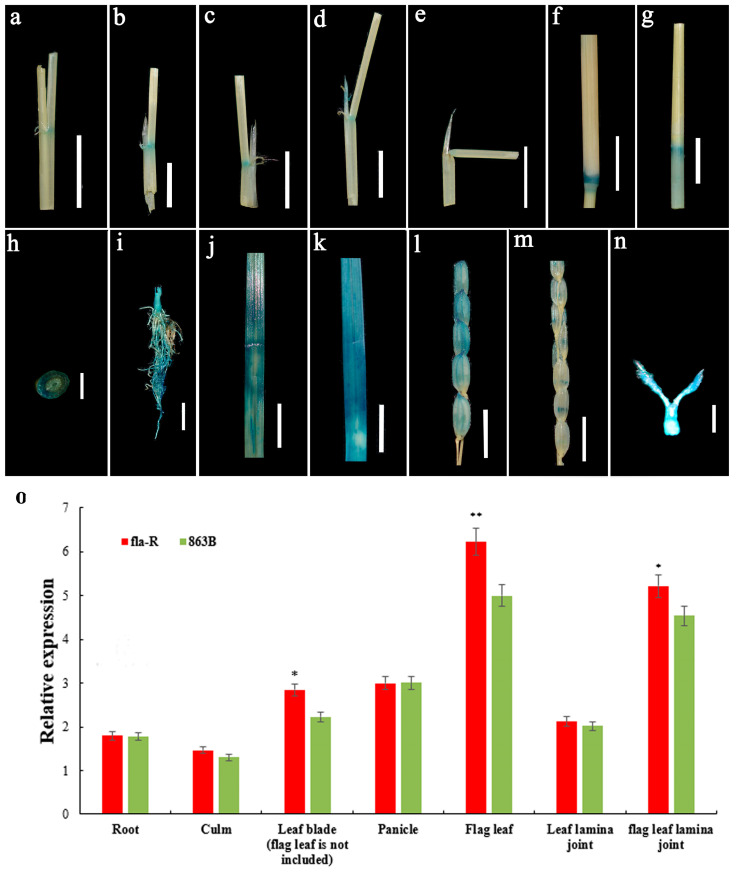
*FLA1* exhibits a relatively high expression level in the flag leaf. (**a**–**n**) GUS stain situation in different tissues of *PRO_FLA1_^Nipponbare^* plants; (**a**) leaf lamina joints; (**b**–**d**) flag leaf lamina joints at stages 6 (**b**), 7 (**c**,**d**), and 8 (**e**) of the young panicle differentiation process, respectively; (**f**) node; (**g**) internode; (**h**) cross section of node; (**i**) root; (**j**) leaf blade; (**k**) flag leaf blade; (**l**) young panicle before flowering; (**m**) young panicle after flowering; (**n**) stigma ((**1**–**m**): bars = 1 cm; (**n**): bar = 100 μm); (**o**) relative expression of *FLA1* in different tissues. * represents significant differences at *p* < 0.05 probability level. ** represents significant differences at *p* < 0.01 probability level.

The spatial and temporal expression of *FLA1^fla-R^* was investigated to study how it functions at the molecular level. *FLA1* transcripts were detected in nearly all organs investigated via RT-qPCR (Figure 9o). Notably, *FLA1* exhibited the highest expression levels in flag leaves and flag leaf lamina joints, with the expression level in fla-R being significantly higher than that in 863B (*p* < 0.01). Meanwhile, *FLA1* also showed relatively high expression levels in other leaves (excluding flag leaves) and leaf sheaths, but low expression levels in roots and stems (Figure 9o). Consistent with the RT-qPCR results, GUS activity was predominantly detected in the vascular bundles of roots, stems, flag leaves, non-flag leaves, flag leaf lamina joints, young panicles, and spikelets (Figure 9a–n). Furthermore, strong *FLA1* signals were observed in tissues with vigorous growth, such as the internode elongation zone (Figure 9f,g,i). Additionally, the expression signal in flag leaf lamina joints was stronger than that in non-flag leaf lamina joints (Figure 9a,c). The GUS signal showed a decreasing trend from the sixth to the eighth panicle differentiation stages (Figure 9b,d,e). The high expression of *FLA1* in flag leaves and flag leaf lamina joints suggests that it may play an active role in FLA enlargement during panicle differentiation.

### 2.6. FLA1^fla-R^ Promotes GA_3_ Synthesis by Promoting the Expression of CLA1 and GA20ox2

**Figure 10:**
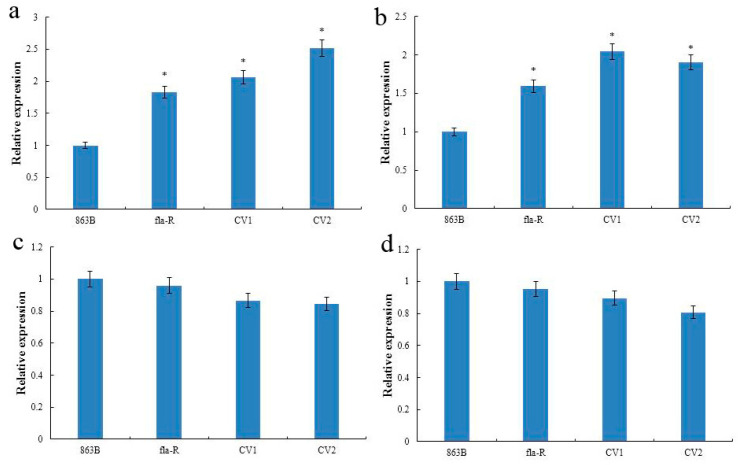
Relative expressions of four key genes related to GA synthesis. Relative expression of *CLA1* (**a**), *GA20ox2* (**b**), *GA1* (**c**), and *KAO* (**d**) in 863B, fla-R, and transgenic *FLA1^fla-R^* complementary lines (CV1 and CV2). * represents significant differences at *p* < 0.05 probability level compared with CK and 863B.

In order to investigate whether *FLA1^fla-R^* can affect GA_3_ synthesis, six key genes for GA synthesis (*CLA1*, *GA1*, *GA20ox2*, *GA2*, *KAO*, and *GA20ox1*) were selected. qRT-PCR analysis was performed on the flag leaf and flag leaf lamina joints of the 7th stage of panicle differentiation (863B, fla-R, *CV-FLA1^863B^*). It was found that the relative expression of *GA2* and *GA20ox1* in flag leaf and flag leaf lamina joints is very low, or even nearly non-expressive; there was no significant difference in the expression levels of *GA1* and *KAO* among the three materials; the expression levels of *CLA1* and *GA20ox2* in fla-R and *CV-FLA1^863B^* were significantly higher than 863B (*p* < 0.05) (Figure 10). This shows that *FLA1^fla-R^* may increase the amount of GA synthesis by promoting the expression of *CLA1* and *GA20ox2*, leading to the flag leaf inclination.

### 2.7. FLA1 Interacts with the Rice Auxin-Repressed Protein (ARP1)

**Figure 11:**
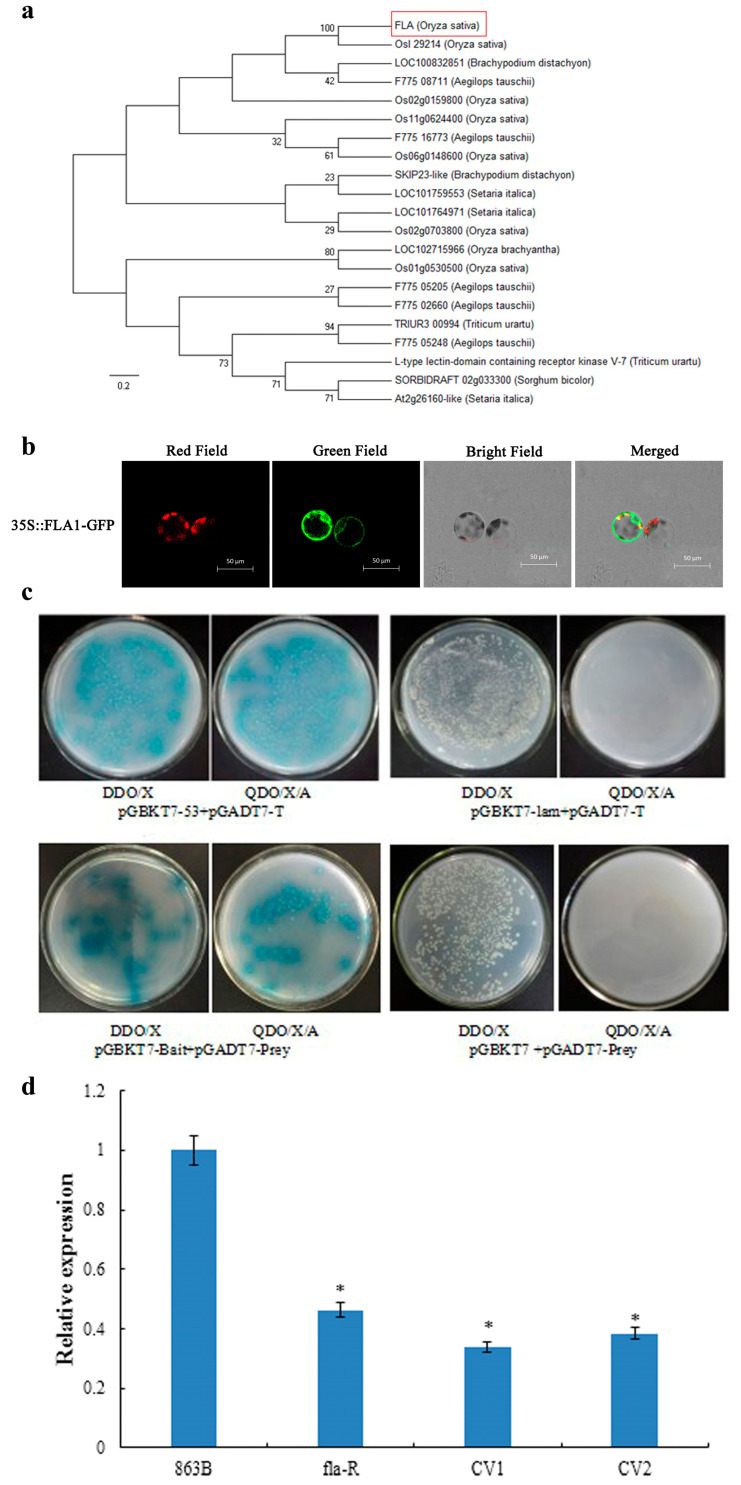
FLA1 interacts with the rice auxin-repressed protein (ARP1). (**a**) Phylogenetic tree of FLA1 and its homologous proteins from different plant species. The rice FLA1 protein is highlighted in red; (**b**) subcellular localization of FLA-GFP fusion protein in rice protoplasts; the *35S::FLA1-GFP* fusion expression vector was utilized for subcellular localization analysis; the panels display signals under four visualization modes: Red Field (red fluorescent signal, typically corresponding to a specific organelle marker), Green Field (green fluorescent signal derived from the FLA1-GFP fusion protein), Bright Field (morphology of the cells), and Merged (superimposed image of the red, green, and bright field signals); the scale bar in each panel represents 50 μm; the merged image suggests the subcellular compartmentalization of FLA1-GFP; (**c**) yeast two-hybrid assay for screening FLA1-interacting proteins; this assay screened for FLA1-interacting proteins from a Y2H library using FLA1 as the bait, with the bait vector co-transformed into Y2H Gold yeast cells alongside library-derived *pGADT7-Prey* clones (and *pGBKT7*+*pGADT7-Prey* as a control), where the top-left panel is the positive control (Y2HGold [*pGBKT7-53*]+Y187 [*pGADT7-T*]) and the top-right panel is the negative control (Y2HGold [*pGBKT7*-lam]+Y187 [*pGADT7*-T]); transformed cells plated on DDO/X and QDO/X/A selective media showed blue colony growth of *pGBKT7*-Bait +*pGADT7*-Prey (consistent with the positive control), indicating a specific interaction, while other controls exhibited expected growth patterns; (**d**) relative expression of *ARF7* in 863B, fla-R, CV1, and CV2. * represents significant differences at *p* < 0.05 probability level.

Phylogenetic analysis revealed that a homologous protein of FLA1 was detected in Artemisia (*Aegilops tauschii*), Sorghum (*Sorghum bicolor*), and Brachypodium distachyon (*Brachypodium distachyon* (L.) *Beauv.*), among other species (Figure 11a). Subcellular localization revealed that the FLA1 protein was located in the cell membrane, nucleus, and chloroplast (Figure 11b). A cDNA library was constructed using flag leaf and flag leaf lamina joints of fla-R. The full-length CDS sequence of *FLA1* was ligated into the *pGBKT7* vector to screen the cDNA library. As shown in Figure 11c, the selected prey protein interacts with the bait protein. Sequencing analysis revealed that the gene encoding this prey protein may correspond to the auxin inhibitory protein (ARP1) encoded by *Os11g0671000*. Moreover, the RT-qPCR analysis of *ARF7* on the flag leaf and flag leaf lamina joints of the 7th stage of ear differentiation (863B, fla-R, and *CV-FLA1^863B^*, the main period of flag leaf lamina joint change). The relative expression of *ARF7* in fla-R and *CV-FLA1^863B^* was significantly lower than that of 863B (*p* < 0.05) (Figure 11d). This shows that after the interaction of FLA1 and ARP1, the change of ARP1 causes *ARF7* to down-regulate expression, release the plant’s inhibition of GA, and promote GA synthesis. Auxin response factor (*ARF7*) is an intermediate regulator of IAA and GA_3_, which inhibits GA synthesis and signal transduction [44]. In this study, the relative expression of ARF7 in fla-R and *CV-FLA1^863B^* was significantly lower than that of 863B (Figure 11d). However, more multi-dimensional protein experiments are still needed to further verify that ARP1 regulates the angle change of rice FLA by interacting with FLA1 to affect GA synthesis.

### 2.8. FLA1 Can Improve the Outcrossing Rate

**Figure 12:**
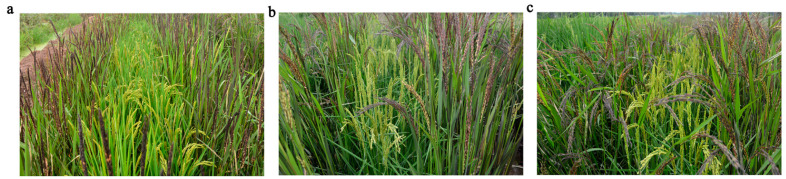
Scene of F_1_ hybrid seed production for three parental combinations in an experimental field. (**a**): 863B/purple rice; (**b**): fla-R/purple rice; (**c**): *CV-FLA1^863B^*/purple rice.

The potential of *FLA1* for hybrid rice seed production was evaluated by the frequency of purple offspring plants (Figure 12). F_1_ seeds (10,000 seeds) were randomly planted in the sowing trays to investigate the frequency of purple leaves in rice. It was found that there were 1032 purple-leaved rice in the F_1_ progeny of small FLA material (863B) and purple rice, and the outcrossing seeding rate was 10.32%. The number of purple leaf rice in the F_1_ of the combination of large FLA materials (fla-R, *CV-FLA1^863B^*, and purple rice) was 1992 and 1894, respectively. The outcrossing seed setting rates were 19.92% and 18.94%, respectively. As a result, Fla-R and *CV-FLA1^863B^* increased the outcrossing setting rate by 9.6% and 8.62%, respectively, compared to 863B.

## 3. Discussion

### 3.1. FLA1 Regulates Rice Flag Leaf Angle by Enhancing GA_3_ Synthesis Through Interaction with ARP1

A core innovation of this study is identifying *FLA1* (*LOC_Os08g31690*), an F-box ubiquitin ligase-encoding gene, as a key regulator of flag leaf angle (FLA) via a gibberellin (GA_3_)-dependent mechanism, which is distinct from the well-documented brassinosteroid (BR)- or auxin (IAA)-dominant regulatory patterns. Notably, RM6215—an FLA-associated marker identified by Dong et al. (2018) through a genome-wide association study—serves as the left flanking marker of the *qFla8-2* locus (interval: RM6215-RM8265) and is tightly linked to *FLA1*. This finding, consistent with the results reported in [45], further verifies that *FLA1* is a functional gene governing FLA and validates the accuracy of our fine-mapped locus, laying a solid foundation for subsequent gene cloning and functional characterization.

The FLA1 protein contains an F-box domain, belonging to the F-box ubiquitin ligase protein family. F-box proteins are characterized by 14–50 highly conserved amino acids, with a C-terminal secondary structure responsible for substrate recognition [46]. Based on the C-terminal secondary structure, F-box proteins are classified into three subfamilies: FBXL (with leucine-rich repeats at the C-terminus), FBXO (with no obvious C-terminal structure), and FBXW (with WD repeats at the C-terminus) [46]. Currently, hundreds of F-box proteins have been identified in plants, whose primary function is to mediate ubiquitination and subsequent hydrolysis of target proteins, thereby participating in various plant physiological regulatory processes, including hormone regulation, light signal transduction, and floral organ development [47]. In terms of hormone regulation, F-box proteins play pivotal roles in GA and BR signaling pathways. Plants contain nuclear protein factors (DELLAs) that act as negative regulators in GA signaling [48]. For example, in the GA-insensitive *Arabidopsis* gai mutant, F-box proteins mediate the degradation of GAI and RGA, resulting in the loss of GA responsiveness [49,50]. Additionally, the C-terminal GRAS domain of *Arabidopsis* DELLA proteins interacts with the N-terminal domain of the BR transcription factor BZR via the leucine heptad repeat 1 (LHR1), thereby inhibiting BZR binding to its target genes and interfering with BR signal transduction [51,52,53]. Beyond hormone regulation, F-box proteins such as ZEITLUPE (ZTL), LOV kelch protein (LKP), and FLAVIN-BINDING, KELCH REPEAT, F-BOX1 (FKF1) also play crucial roles in regulating plant circadian rhythms; for instance, circadian clock disruption was observed in *Arabidopsis* lkp2 overexpression mutants [54]. In this study, amino acid sequence alignment between 863B and fla-R revealed two amino acid mutations (P→L, G→A) in the F-box domain of FLA1 (Figure 6e). NCBI protein structure prediction indicated that these two amino acids are located in the conserved region of the F-box domain, and their mutations may lead to functional alterations—potentially accounting for the FLA differences between fla-R and 863B. Consistent with the functional characteristics of F-box proteins, yeast two-hybrid assays identified ARP1 as an interacting protein of FLA1. ARP1 is an auxin-inhibiting protein; binding to FLA1 may trigger its hydrolysis, thereby relieving the inhibition of auxin [55,56]. Additionally, the auxin response factor *ARF7* acts as an intermediate regulator of IAA and GA_3_ signaling, inhibiting GA synthesis and signal transduction [44]. In this study, the relative expression of *ARF7* in fla-R and *CV-FLA1^863^ᴮ* was significantly lower than that of 863B (Figure 11d). This suggests that after FLA1 interacts with ARP1 and relieves auxin inhibition, the expression level of *ARF7* is reduced, further releasing the inhibition of GA synthesis and signal transduction to promote flag leaf inclination. However, the specific molecular link between ARP1 degradation and *ARF7* downregulation remains to be clarified, and additional experiments (e.g., in vivo ubiquitination assays and *ARF7* promoter activity analysis) are needed to validate this regulatory relationship.

Notably, auxin can promote the transcription of genes involved in the GA synthesis pathway [57,58,59]. For example, in peas, auxin enhances GA20-oxidase activity and inhibits GA2-oxidase activity, thereby promoting the conversion of GA_20_ to the active GA form (GA_1_) and boosting GA synthesis [59,60,61]. Consistent with this, *FLA1* modulates FLA by specifically enhancing GA_3_ synthesis, a regulatory feature distinct from prior studies where BR homeostasis or IAA signaling were the primary drivers of leaf angle changes [12,17,18,21]. Endogenous hormone assays confirmed that fla-R (carrying the favorable *FLA1^fla-R^* allele) had a significantly higher GA_3_ content (0.8 ng/g) in flag leaf lamina joints than 863B (0.09 ng/g, *p* < 0.01), with no significant differences in IAA or BR contents (Supplementary Table S2). Exogenous GA_3_ (10 μM) significantly increased FLA in both 863B and fla-R, while BR (1 μM) and IAA (20 μM) had no obvious effects on FLA (Figure 5), confirming GA_3_ as the primary hormone mediating *FLA1*-dependent FLA enlargement. RT-qPCR further verified that *FLA1^fla-R^* upregulates the expression of GA synthesis key genes *GA20ox2* in flag leaf lamina joints (Figure 10), directly promoting GA_3_ biosynthesis [59,61]. *CLA1* encodes 1-deoxy-D-xylulose 5-phosphate synthase. On the one hand, this synthase participates in the biosynthesis of gibberellin (GA) precursors via the 2-C-methyl-D-erythritol 4-phosphate (MEP) pathway [62,63]; on the other hand, studies have demonstrated that it can respond to GA induction [63,64]. Therefore, we propose that *CLA1* is a gene associated with GA biosynthesis. Moreover, the expression level of *CLA1* was significantly higher in *CV-FLA1^863B^* and fla-R than in 863B (*p* < 0.05), indicating that there may be an association between *FLA1* and *CLA1* at the transcriptional level, which in turn indirectly affects GA biosynthesis—a hypothesis that requires further experimental validation in future research.

**Figure 13:**
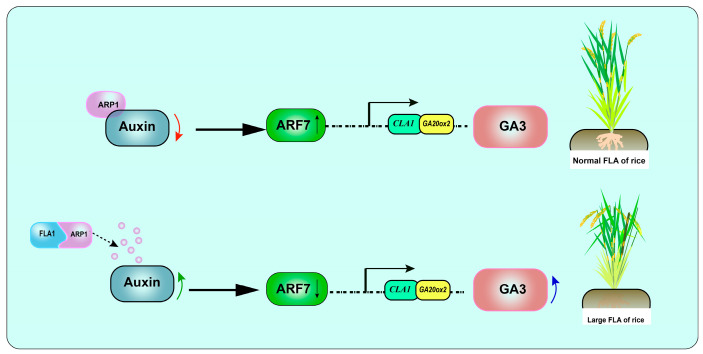
Hypothetical model of the function of *FLA1* in terms of the flag leaf angle. This figure depicts the *FLA1*-mediated molecular pathway underlying FLA variation in rice, presenting two scenarios: the upper pathway (normal FLA) involves ARP1 suppressing auxin activity, which results in elevated *ARF7* transcription; *ARF7* then inhibits *CLA1* and *GA20ox2* via a putative regulatory relationship, leading to a typical rice flag leaf angle, while the lower pathway (large FLA) features FLA1 mediating ARP1 degradation to enhance auxin activity and reduce *ARF7* transcription; reduced *ARF7* thus relieves its inhibition on *CLA1* and *GA20ox2*, promoting GA_3_ accumulation and consequently an enlarged rice flag leaf angle. Note: dashed lines represent putative regulatory relationships; red arrows indicate reduced activity; green arrows indicate increased activity; blue arrows indicate increased content; black upward arrows (↑) denote elevated transcription levels; black downward arrows (↓) denote reduced transcription levels.

Cytologically, FLA enlargement in fla-R arises from reduced adaxial cell layers and elongated abaxial longitudinal cells in lamina joints (Figure 4, Figure 7 and Figure 8)—consistent with conserved cytological features of leaf angle regulation [12,22,24]. However, *FLA1*’s regulatory target is unique: previous studies linked similar cytological changes to BR-mediated cell proliferation or GA-dependent cell elongation, while *FLA1* coordinates both processes via GA_3_ synthesis, acting as an upstream regulator to modulate downstream cytological events [12,22]. Collectively, our results support a model where FLA1 (with functional mutations in its F-box domain) interacts with ARP1 to relieve auxin inhibition, upregulates GA synthesis-related genes to enhance GA_3_ accumulation, and reduces *ARF7* expression to release GA signaling inhibition, ultimately promoting FLA enlargement (Figure 13).

### 3.2. FLA1 Enables High Efficiency Mechanized Hybrid Rice Seed Production via Flag Leaf Angle Optimization

A critical advantage is *FLA1*’s specificity in regulating FLA: fla-R exhibits a significantly enlarged FLA (90–110°) compared to 863B (15–25°), but no significant differences in the second/third leaf angles, 1000-grain weight (Figure 3). Notably, fla-R shows a significantly higher plant height than 863B (Figure 3d). This phenotypic difference is likely attributed to the pleiotropic effect of *FLA1*: as *FLA1* is involved in mediating GA and IAA signaling pathways—both of which regulate cell number and elongation—its functional variation may simultaneously modulate flag leaf angle and plant height. Nevertheless, this plant height difference does not compromise the application value of *FLA1* in hybrid rice seed production, as the core agronomic traits related to yield remain unaffected. This specificity, derived from *FLA1*’s high expression in flag leaf lamina joints (Figure 9), avoids the drawbacks of previously reported large-leaf-angle mutants/QTLs that affect multiple traits. For hybrid rice seed production, the female parent with large FLA eliminates the need for manual flag leaf cutting (a labor-intensive step to improve pollination efficiency), while the F_1_ hybrid retains normal plant type (due to the recessive inheritance of large FLA), preventing yield losses from excessive leaf inclination [6,7,65]. This trait optimization ensures that the application of *FLA1* is not only technically feasible but also adaptable to practical production scenarios.

Field experiments confirmed that fla-R and *FLA1-complemented lines* (*CV-FLA1^863B^*) had outcrossing seed-setting rates of 19.92% and 18.94%, respectively—9.6% and 8.62% higher than that of 863B (10.32%)—when crossed with purple-leaf male parents (Figure 12). This improvement stems from the enlarged FLA reducing pollination barriers between male and female parents. Unlike chemical regulation (e.g., GA_3_ spraying), which requires precise timing and dosage control, molecular breeding using *FLA1* provides a stable, environment-friendly solution for mechanized seed production [43]. Moreover, the stable inheritance of the *FLA1^fla-R^* allele ensures consistent performance in subsequent breeding generations, further enhancing its practical value. Additionally, *FLA1* is highly compatible with japonica hybrid rice breeding systems. Japonica hybrid rice, widely cultivated in Jiangsu, Zhejiang, and other regions of China, has long faced challenges in mechanized seed production due to the small FLA of female parents [6,36]. The introduction of *FLA1^fla-R^* into japonica sterile lines or maintainer lines can directly solve this problem, providing a practical genetic tool for optimizing plant type in hybrid rice seed production. Furthermore, combined with existing molecular breeding technologies, *FLA1* can be rapidly integrated into elite rice germplasm resources, accelerating the breeding process of mechanization-adapted hybrid rice varieties.

### 3.3. Research Limitations and Future Directions

While this study clarifies the core regulatory mechanism and application value of *FLA1*, several key aspects require further exploration. First, the in vivo validation of the FLA1-ARP1 interaction and its downstream effects remains incomplete. Although yeast two-hybrid assays confirmed their interaction, follow-up experiments such as co-immunoprecipitation, bimolecular fluorescence complementation, and in vivo ubiquitination assays are needed to verify the binding specificity and degradation dynamics of ARP1 by FLA1. Second, the specific molecular link between ARP1 degradation and *ARF7* downregulation needs to be elucidated, which will help clarify the complete regulatory relationship of *FLA1* in GA synthesis. Third, the functional impact of the two amino acid mutations (P→L, G→A) in FLA1’s F-box domain requires direct verification; site-directed mutagenesis experiments can clarify whether these mutations are the direct cause of *FLA1*’s functional alteration. Fourth, the generalization of *FLA1*’s function in indica rice warrants investigation. Given the differences in plant type and hormone sensitivity between indica and japonica rice, validating *FLA1*’s regulatory effect in indica backgrounds will help expand its application scope in hybrid rice breeding. Future research focusing on these aspects will further improve the understanding of *FLA1*’s regulatory network and enhance its breeding application value, providing stronger support for mechanized hybrid rice seed production.

## 4. Materials and Methods

### 4.1. Plant Materials and Growth Conditions

The plant materials employed in this study were as follows: 863B, a maintainer line of the commercial japonica hybrid rice cultivar 86-you-8hao that is widely cultivated in Jiangsu Province, China, with a mean flag leaf angle (FLA) of 20° (range: 15–25°); A7444, a japonica landrace accession from the germplasm collection of the Taihu Lake Basin, China, with a mean FLA of 140° (range: 120–160°) [36,66]; fla-R, a near-isogenic line (NIL) harboring the large-FLA allele from A7444 in the 863B genetic background (Figure 2); and a BC_3_F_4_ single locus segregating population consisting of 4382 plants. All experimental materials were grown annually at the Jiangpu Experimental Station of Nanjing Agricultural University, Nanjing (32°07′ N, 118°64′ E), Jiangsu Province, China, under natural field conditions. The key cultivation parameters were as follows: natural photoperiod, average growth temperature of 22–30°C (consistent with local rice-growing season), basal compound fertilizer (N:P:K = 15:15:15) applied at 450 kg/ha, urea topdressing (150 kg/ha) at tillering and panicle initiation stages, and conventional flood-intermittent irrigation (3–5 cm water layer initially, then alternating wet and dry until maturity). In 2014, 32 plants each of 863B and A7444, and 4382 plants of the single locus segregating population were planted. In 2015, T_1_ transgenic lines were grown in an isolated plot at the same station. In 2016 and 2017, T_2_ and T_3_ transgenic complementary lines and overexpression lines were planted in isolated plots at this station. Seedlings were transplanted singly per hill with a spacing of 17 cm between plants and 20 cm between rows, following the above natural field cultivation parameters and routine field management. The outcrossing seed-setting rate evaluation experiment for each female parent (863B, fla-R, and transgenic lines) was conducted as follows: in each seed production plot, 2 rows of the female parent were transplanted in the center, with 2 rows of the male parent (purple-leaf rice) on each side. Seed production plots for the combinations (863B/purple rice, fla-R/purple rice, and transgenic lines/purple rice) were mutually isolated. All seeds used in this study were preserved and multiplied by the corresponding author at the State Key Laboratory of Crop Genetics and Germplasm Enhancement, Nanjing Agricultural University.

### 4.2. Character Investigation and Plant Hormone Treatment

FLA was measured using a protractor when the main stem panicle emerged 10 cm above the flag leaf lamina joint (Supplementary Figure S1). For each accession, the average FLA of 10 individual plants was taken as the phenotypic value for subsequent analysis. For the field hormone treatment assay, 10 main stem panicles at the third young panicle differentiation stage were selected from each strain (fla-R, A7444, and 863B) for phytohormone application. The treatment concentrations were as follows: 1 µM 24-eBL (a highly active brassinosteroid, E1641; Sigma, Kanagawa, Japan), 10 µM GA_3_ (gibberellic acid, G7645; Sigma, Kanagawa, Japan), and 20 µM IAA (I2886; Sigma, Kanagawa, Japan) [22]. Distilled water treatment was used as the control.

### 4.3. Observation of Cell Morphology

Flag leaf lamina joints were isolated from near-isogenic lines (fla-R, 863B) and landrace A7444 at the sixth, seventh, and eighth young panicle differentiation stages. Two tissue sectioning methods were employed in this study: (1) paraffin sectioning, performed as modified from Yang (2006) [67]. The specific steps are as follows: fix the isolated lamina joints in 4% paraformaldehyde (*w*/*v*, dissolved in 0.1 M phosphate-buffered saline, PBS, and pH 7.4) at 4 °C for 12–16 h; dehydrate the tissues through a graded ethanol series (70%, 85%, 95%, 100%, and *v*/*v*) for 30 min each; clear with xylene for 2 × 30 min; infiltrate and embed in paraffin wax (melting point 56–58 °C) at 60 °C; cut into 5–8 μm sections using a rotary microtome; mount sections on poly-L-lysine-coated glass slides, bake at 60 °C for 2 h to adhere firmly; deparaffinize with xylene, rehydrate through a reversed ethanol series, and stain with hematoxylin-eosin (H&E) or toluidine blue O for structural observation; (2) Frozen sectioning: Flag leaf lamina joints were immersed in OCT embedding medium for 20 min, then transferred to the sample stage of a Leica cryostat (CM1950, Wetzlar, Germany). Samples were equilibrated in the cryostat chamber (adjusted to the specified chamber and head temperatures) for 20–40 min, followed by sectioning into 10–15 μm slices for structural observation. All tissue sections were examined using a fluorescence microscope (BX53, Olympus, Tokyo, Japan).

### 4.4. QTL Mapping of FLA1

A genetic linkage map of the near-isogenic line (NIL) population was constructed using 401 simple sequence repeat (SSR) markers via MapMaker/EXP 3.0 software [68]. QTL analysis was conducted via QTL Mapper 2.0 combined with the time-course QTL mapping approach. For fine mapping, 4382 plants with a large flag leaf angle (FLA > 85°) were selected from the single locus segregating population.

### 4.5. Gene Cloning, Vector Construction, and Plant Transformation

The *FLA1* gene locus (*LOC_08g31690*) was retrieved from the *Oryza sativa* ssp. japonica (Nipponbare) genome annotation databases (Gramene and NCBI). Gene-specific primers flanking the full-length of *FLA1* were designed using Primer Premier 5.0 software (primer sequences are provided in Supplementary Table S4), and the full-length *FLA1* CDS was amplified from the cDNA template of flag leaf lamina joints of fla-R using Q5 High-Fidelity DNA Polymerase (New England Biolabs, Ipswich, MA, USA). For the genetic complementation test, the 2.1 kb genomic DNA fragment containing fla-R (Flag leaf angle recessive) was amplified using Q5 High-Fidelity DNA Polymerase (New England Biolabs, Ipswich, MA, USA) and then cloned into a *pCAMBIA1301* binary vector (Bio Run, Nantong, China) using an In-Fusion Advantage PCR Cloning Kit (Clontech, Shiga, Japan). The resultant plasmid was transformed into *Agrobacterium tumefaciens* strain EHA105, which was then introduced into 863B via *A. tumefaciens*-mediated transformation. The full-length gene controlled by the Cauliflower mosaic virus 35S promoter was cloned into a *pCAMBIA1301* plant binary vector to generate *OE-FLA1^fla-R,^* which was subsequently introduced into fla-R by *A. tumefaciens*-mediated transformation as reported previously [69]. At least 30 transgenic events were produced for each construct.

### 4.6. GUS Staining

The promoter region of 2 kb upstream of the gene FLA1 was cloned into the vector *pCAMBIA1304* to create *PROFLA1*: GUS, and the restriction sites were *KpnI* and *SpeI*. The resultant plasmid was transformed into A. tumefaciens strain EHA105, which was then introduced into Nipponbare. After obtaining T_2_ transgene positive plants, GUS staining was performed as described by Jefferson et al. (1987) [70]. Various tissues or hand-cut sections of *PRO_FLA_*: GUS T_2_ generation transgenic plants were incubated in GUS staining solution. Images were captured directly or with a stereomicroscope.

### 4.7. Subcellular Localization of the FLA1 Protein

The CDS of gene *FLA1* was cloned into the vector *pCAMBIA1304* to obtain the FLA1-GFP fusion protein vector started by the *Cauliflower mosaic virus 35s* promoter. The fusion construct was transformed into rice leaf protoplasts via polyethylene glycol. The cells were then examined with a confocal fluorescence microscope, and the fluorescence was observed using a Leica TCS-SP5 confocal microscope (Wetzlar, Germany).

### 4.8. Construction of cDNA-AD Library and Yeast Assays

A cDNA-AD library of rice flag leaves and flag leaf lamina joints was constructed by Genecreate Company (Wuhan, China). The yeast two-hybrid system was used to perform transcriptional activation assays of FLA1. FLA1 was cloned into the *pGBKT7* vector to generate the recombinant construct *pGBKT7*-FLA1 (BD-FLA1), which was then used to screen the cDNA-AD library. Hybridization mixtures of cDNA and BD-FLA1 were plated on QDO/A selective medium. Y2H Gold (*pGBKT7*-53) and Y187 (*pGADT7*-T) were cotransformed as positive controls, while Y2H Gold (*pGBKT7*-lam) and Y187 (*pGADT7*-T) served as negative controls. Positive single colonies were sequenced, and proteins without frameshifts were selected as candidate interacting proteins. Transcriptional activation ability was evaluated on QDO + X-α-Gal selective medium, and protein-protein interactions were verified on QDO selective medium.

### 4.9. Amino Acid Sequence Alignment and Homology Analysis

The BLASTP search tool (version 2.6.0+) on the NCBI database was used to identify homologous amino acid sequences of *FLA1*. Homologous sequence alignment of the search results was performed using DNAMAN 6.5 software, and phylogenetic tree analysis was conducted with MEGA 5.0 software.

### 4.10. Total RNA Extraction and RT-qPCR Analysis

Total RNA was extracted from various plant tissues using the RNApure Plant Kit (CW0588, Kangwei Century Biotechnology Co., Ltd., Beijing, China). First-strand cDNA was synthesized via reverse transcription of RNA using the 1st Strand cDNA Synthesis Kit (Vazyme, Nanjing, China) following the manufacturer’s protocol. Quantitative real-time PCR (qPCR) was performed on the Roche Applied Science LightCycler 480 system with SYBR Green Master Mix (Vazyme, Nanjing, China) according to the operation manual. The rice 18S rRNA gene was used as an internal control, and the relative transcript levels were quantified using the comparative threshold cycle (Ct) method [71].

### 4.11. Endogenous Hormone Determination

Endogenous hormones (IAA, GA_3_, BR) were quantified in the flag leaves and flag leaf lamina joints of 863B, A7444, and fla-R at the sixth young panicle differentiation stage. The extraction method of endogenous hormones from rice flag leaves was performed as described by Andrew R.S. Ross et al. (2004) [72]. Plant endogenous hormone determination was conducted using liquid chromatography-tandem mass spectrometry (LC-MS/MS) with the following instruments: Agilent 1290 high-performance liquid chromatograph (Agilent Co. Ltd., Santa Clara, CA, USA) and SCIEX-6500 Qtrap (MS/MS) (AB Co. Ltd., Lincolnwood, IL, USA). Sample preparation methods: for IAA, GA_3_, and Zeatin, approximately 1 g fresh rice leaf samples were ground into powder in liquid nitrogen and extracted with 10 mL isopropanol/hydrochloric acid buffer at 4 °C with shaking for 30 min. Then 20 mL of dichloromethane was added, followed by shaking at 4 °C for another 30 min. After centrifugation at 4 °C, 13,000rpm for 5 min, the lower organic phase was collected, dried under nitrogen in the dark, dissolved in 200 μL methanol (0.1% formic acid), and filtered through a 0.22 μm membrane for detection. For BR, approximately 0.5g rice samples were ground into powder in liquid nitrogen, extracted with 10 mL pre-cooled 80% methanol at 4 °C for 2h. After centrifugation (4 °C, 10,000 rpm, 5min), the supernatant was collected, purified by Bond Elut and Strata-X columns (eluted with 3mL methanol sequentially), dried under nitrogen, dissolved in 200 μL methanol, and filtered through a 0.22 μm membrane for detection. Recovery rate validation: the average recovery rates of IAA, GA_3_, Zeatin, and BR were 88.5–92.3% and 89.2–93.5%, respectively (RSD < 5%), verifying the reliability of sample preparation. Liquid chromatography conditions: Poroshell 120 SB-C18 reversed-phase column (2.1 × 150mm, 2.7 μm); column temperature: 30 °C; mobile phase: A (methanol/0.1% formic acid) and B (water/0.1% formic acid); elution gradient: 0–2 min, 20% A; 2–4 min, linear gradient to 50% A; 4–10 min, linear gradient to 80% A; 10–11 min, 80% A; 11.1 min, immediate decrease to 20% A; 11.1–15 min, 20% A; injection volume: 2 μL. Quantitative calibration curve information: standard solutions of IAA, GA_3_, and Zeatin (0.1–200ng/mL) were prepared with methanol (0.1% formic acid), and their regression equations, correlation coefficients (r), and weighting are: IAA: y = 5.36644e4x + 168.90283 (r = 0.99568); GA_3_: y = 10871.45494x + 1254.95765 (r = 0.99403); Zeatin: y = 4.66928e5x-182.79052 (r = 0.99076) (weighting: 1/x^2^). For BR, standard solutions (0.5–20ng/mL) were prepared with methanol, and the calibration curve equation was y = 529.60423x + 46.26495, with a correlation coefficient R^2^ = 0.99773 (weighting: 1/x^2^). Mass spectrometry conditions: curtain gas: 15 psi; spray voltage: 4500 V; nebulizer gas pressure: 65psi; auxiliary gas pressure: 70 psi; ion source temperature: 400 °C.

## 5. Conclusions

We successfully cloned and functionally characterized the *FLA1* gene (mapping to a major-effect QTL) that controls rice flag leaf angle (FLA); comprehensive analyses integrating cell morphology, exogenous hormone treatments, genetics, and molecular biology clarified FLA’s critical role in regulating FLA and its association with gibberellin (GA) signaling pathways, demonstrating that GA modulates FLA by altering cell layer number and cell length with *FLA1* as a key regulator in this process, while *FLA1*’s constitutive expression and localization in both cell membrane and nucleus provide clues for its molecular function, which was further validated by genetic complementation and overexpression assays to be sufficient for controlling FLA; notably, the *FLA1* allele from fla-R accession enhances GA biosynthesis by upregulating *CLA1* and *GA20ox2* expression, revealing the molecular mechanism of *FLA1* regulating FLA via GA biosynthesis, and yeast two-hybrid assays identified the interaction between FLA1 and auxin-repressed protein 1 (ARP1), suggesting potential crosstalk between GA and auxin signaling pathways in FLA regulation and laying a foundation for exploring the complex hormone regulatory network underlying FLA determination; practically, our findings highlight *FLA1*’s application value in crop breeding, as its molecular manipulation can optimize FLA to resolve flag leaf shearing during mechanized F_1_ hybrid rice seed production without compromising yield, enriching our understanding of rice plant architecture regulation mechanisms and providing valuable genetic resources and technical strategies for facilitating mechanized F_1_ hybrid rice seed production, which is of great significance for improving rice production efficiency and sustainability.

## Data Availability

The original contributions presented in this study are included in the article/Supplementary Material. Further inquiries can be directed to the corresponding author.

## Supplementary Materials

The following supporting information can be downloaded at: https://www.mdpi.com/article/10.3390/plants15030446/s1, Figure S1. Operations of hormone application and flag leaf angle measurement in paddy field. (a) Hormone application; (b) flag leaf angle measurement. Figure S2. 863B/A7444 combination BC_1_F_1_ population SSR marker linkage map and location of flag leaf angle QTL on chromosome. Figure S3. Genetic analysis of the *qFla8* locus on chromosome 8. Figure S4. Patterns of leaf occipital shape, cross section of leaf occipital lobe, and number of cell layers at the distal end of leaf stalk of 863B, A7444, fla-R. (a–c) are 863B, fla-R, A7444 leaf occipital morphological comparisons of young panicle differentiation at sixth stage; (d and e) occipital transection cell morphology in 863B (d) and fla-R (e); (f) abdominal end (ab) leaf occipital transect cells morphology in A7444; (g) adaxial end (ad) leaf occipital transect cells morphology in A7444; (h) statistical comparison of parenchymal cell layer numbers at the adaxial (ad) and abaxial (ab) ends of the flag leaf lamina joint across the three genotypes (a, b, c: bar = 2.5 cm, d, e, f, g: bar = 150 μm). Figure S5. Amino acid sequence alignment of ORF3 (LOC_Os08g3670) and ORF4 (LOC_Os08g3690) between 863B and fla-R. (a) ORF3 (LOC_Os08g3670); (b) ORF4 (LOC_Os08g3690). Figure S6. Amino acid sequence alignment of FLA1 of fla-R and 863. Table S1. Putative QTLs and their additive effects for flag leaf angle in japonica rice BC_1_F_1_ population. Table S2. Detection result of endogenous hormone. Table S3. Predicted genes at the *FLA1* locus; Table S4. Primer sequence designed in this study.

## Abbreviations

The following abbreviations are used in this manuscript:

FLA1	FLAG-LEAF-ANGLE 1
FLA	Flag leaf angle
ARP1	Auxin-repressed protein
BR	Brassinosteroid
QTL	Quantitative trait locus
NIL	Near-isogenic line
fla-R	Flag leaf angle recessive
ORF	Open reading frame
ARF7	Auxin response factor
LC2	Leaf inclination2
IAA	Indole-3-Acetic Acid
GA	Gibberellic Acid
