# Supplementary material for: FLA1, Enhancing GA3 Contents in Flag Leaf Lamina Joint, Increases Flag Leaf Angle to Improve Outcross Rate and Hybrid Rice Seed Production
Source: Plants (Basel). 2026 Jan 31;15(3):446. doi: 10.3390/plants15030446 (PMC12899354; doi:10.3390/plants15030446)
Supplement: Supplementary file 1 [file plants-15-00446-s001.zip › Supplementary Figure S1-6;Table S1-4.pdf]

Supplementary Materials

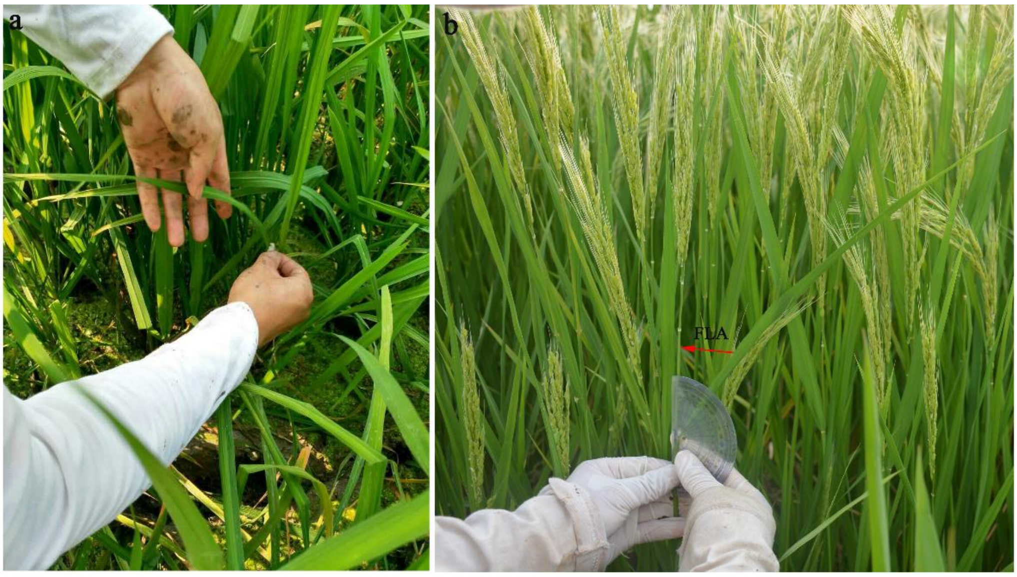

Figure S1. Operations of hormone application and flag leaf angle measurement in paddy field. (a) Hormone application; (b) Flag leaf angle measurement.

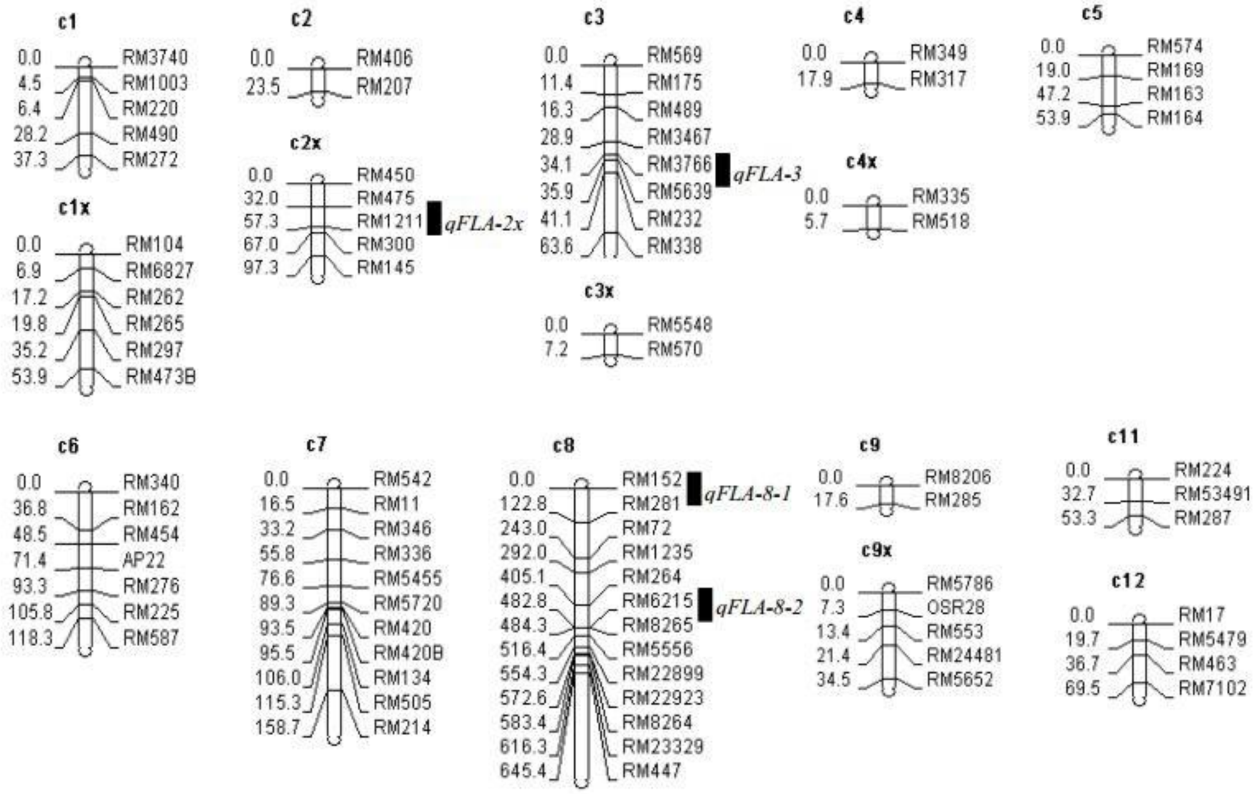

Figure S2. 863B/A7444 combination BC_1F_1 population SSR marker linkage map and location of flag leaf angle QTL on chromosome.

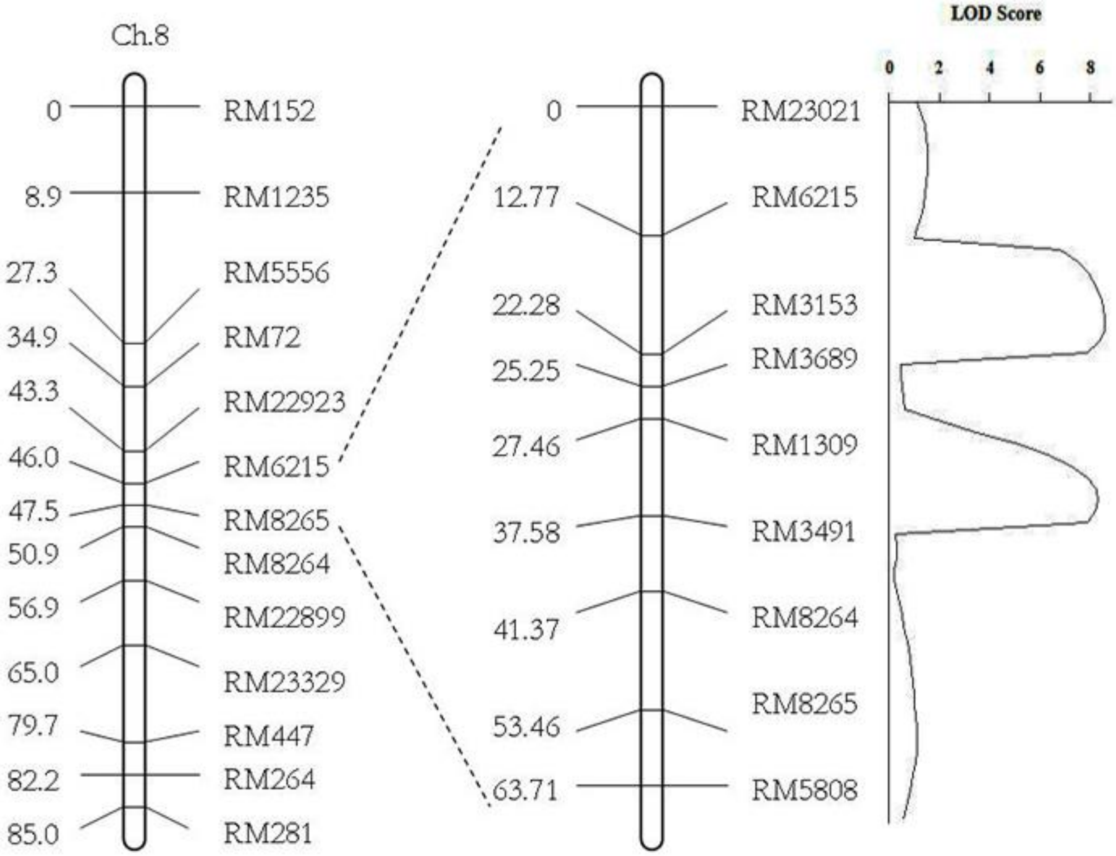

Figure S3. Geneic analysis of *qFla8* locus on chromosome 8.

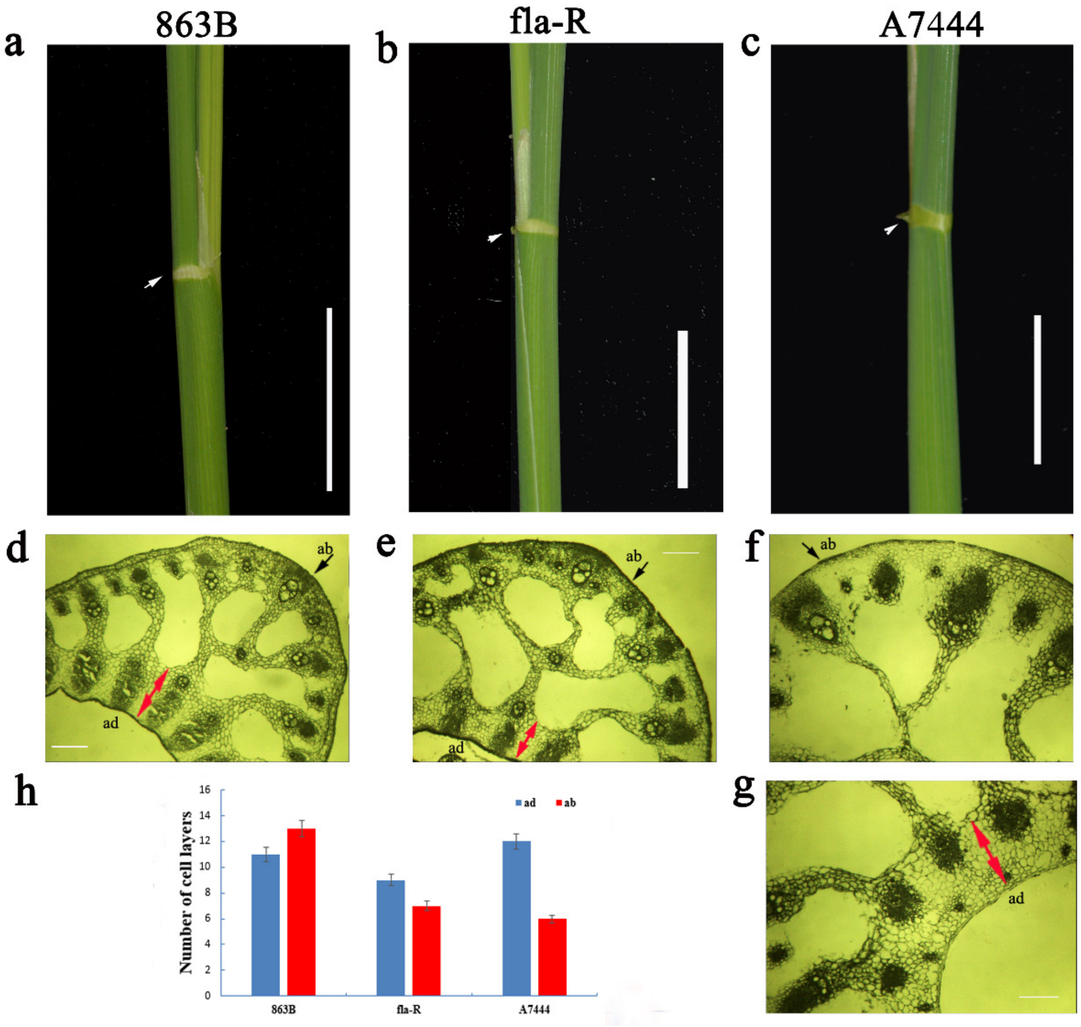

Figure S4. Patterns of leaf occipital shape, cross section of leaf occipital lobe, and number of cell layers at the distal end of leaf stalk of 863B, A7444, fla-R. (a-c) are 863B, fla-R, A7444 leaf occipital morphological comparisons of young panicle differentiation at 6th stage; (d and e) Occipital transection cell morphology in 863B (d) and fla-R (e); (f) Abdominal end (ab) leaf occipital transect cells morphology in A7444; (g) Adaxial end (ad) leaf occipital transect cells morphology in A7444; (h) Statistical comparison of parenchymal cell layer numbers at the adaxial (ad) and abaxial (ab) ends of the flag leaf lamina joint across the three genotypes (a, b, c: bar = 2.5 cm, d, e, f, g: bar = 150 μm).White arrows indicate the flag leaf lamina joint ; red arrows indicate the number of cell layers (ad).

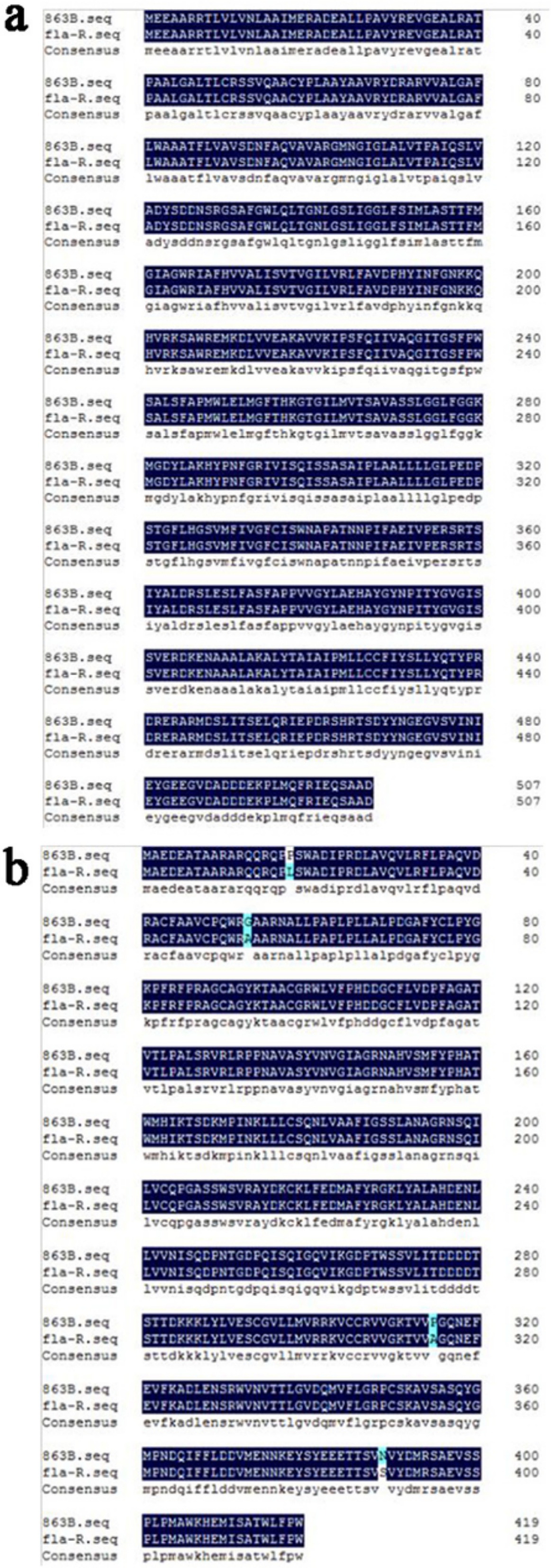

Figure S5. Amino acid sequence alignment of ORF3 (LOC_Os08g3670) and ORF4 (LOC_Os08g3690) between 863B and fla-R. (a) ORF3 (LOC_Os08g3670); (b) ORF4 (LOC_Os08g3690).

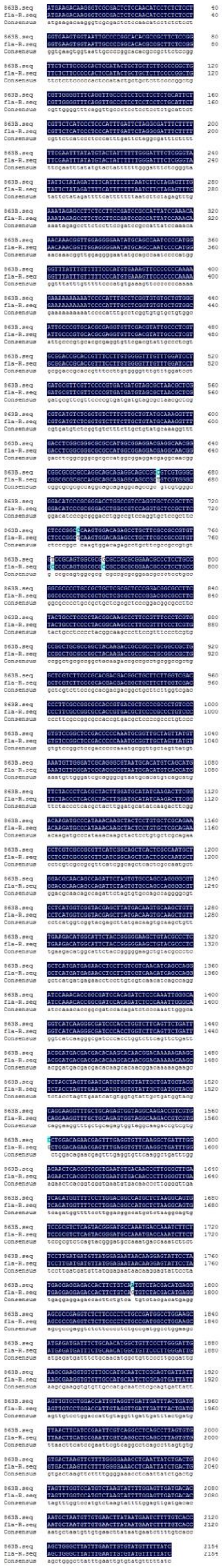

Figure S6. Amino acid sequence alignment of FLA1 of fla-R and 863.

Table S1. Putative QTLs and their additive effects for flag leaf angle in japonica rice BC_1F_1 population

QTL	Marker interval	Distance/cM	Confidence interval	Additive effect	Variance explained/%
qFla2	**RM300**-RM145	35.5	31.2-44.5	-24.65	10.50
qFla8	**RM6215**-RM8265	46.0	34.9-53.9	-23.49	9.59

Note：1) Bold letters indicate the nearer marker to putative QTL;

2) – mean that positive alleles come from A7444.

Table S2. Detection result of endogenous hormone

Hormone	GA_3		IAA		BR	
Material	863B	fla-R	863B	fla-R	863B	fla-R
Detection concentration（ng/L）	0.43	4.40	1.05	1.00	0.35	0.15
Quality factor（g）	0.99	1.10	0.99	1.10	0.60	0.50
Volume factor（mL）	0.2	0.2	0.2	0.2	0.2	0.2
Content（ng/g）	0.09	0.80**	0.21	0.18	0.12	0.06

**：$P < 0.001$

Table S3. Predicted genes at the FLA1 locus

(Predicted gene)	(Locus ID)	(Predicted function)
**ORF1**	LOC_08g31630	Trehalose-phosphatase domain containing Protein
**ORF2**	LOC_08g31660	VQ domain containing protein
**ORF3**	LOC_08g31670	hexuronate transporter
**ORF4**	LOC_08g31690	OsFBX291 - F-box domain containing protein, DUF293 domain

Table S4. Primer sequence designed in this study

Marker	Purpose	Primer-F(5’-3’)	Primer-R(5’-3’)
RM1309	Fine mapping	GAGGACACTGACGACAGCTTGG	CGCGCAAATCATTAAGTTCAGG
RM8264	Fine mapping	TTCTACGGAATTTCTCCCTCTGG	CTAATCAATCTCTCGCGTTCTTGG
RM23071	Fine mapping	GTTCCGCCGTTGAGTGATGACC	TCCTCAGTCCTCCCTCTCCTTCC
RM23065	Fine mapping	CCACGAACTCTCCCTATATCTACTGC	CGTGCACACCTGAAGAGTATGG
InDel 5	Fine mapping	CTCGAGGCGTTCATGGTTACTTCG	TGCGTTGCACTGGGTGATTACG
InDel 7	Fine mapping	GGTGCAAGTAGAAGGAAGGAAGG	AAAGGCAAGCAACGAGAAGACC
InDel 36	Fine mapping	AGACGGAAAGCAGCAAAAGC	GCATGCGTGTCGTTGTGAAT
q-RT1	qRT-PCR	AGGCTTAATCTGTCGCCGTC	GGTGGTCATTACCTCGGCTG
q-RT2	qRT-PCR	GCTGCTGCTACTACTACTGGTC	ACTGGAGACTCCCGCTGTTG
q-RT3	qRT-PCR	ATGTGGCTGGAGTTGATGGG	AGATGGGTCTTCAGGCAAGC
q-RT4	qRT-PCR	GACTTCGGACAAGATGCCCA	CCCTTGATGACCTGCCCAAT
q-RT4-1	qRT-PCR	ATTGGGCAGGTCATCAAGGG	CCCACCGTGAGTTCTCCAAA
18sRNA	qRT-PCR	ATGATAACTCGACGGATCGC	CTTGGATGTGGTAGCCGTTT
S1	Sequence	GGCGCCTCCTCCAACTATAAA	AGTAAGAAGGGAGGGGACGA
S2	Sequence	TTTCCGCAGCGCCTAATCAT	TTGATCGATCTACACAAAGAGCA
S3	Sequence	TGCTCATCGAGCAAAGGTCC	GCTAGCTCGGCTTACAGGAT
S4	Sequence	AACCATGATTGAACGGCCCA	CAACGTTCAGTCCAAACTCCA
hyg	Detection	ACGTGTCGTCCATCACAGTTTGCC	TTCCGGAAGTGCTTGACATTGGGGA
FLA1-Pro	Vector construction	GGACTAGTGAAGCAGGCTCTGTCCACTT	GGGGTACCTAGACGGGCCAGGATGGTAA
FLA1-GFP	Vector construction	CGGCTCGAGATGGCGGAGGACGA	CGGGGTACCATCCCAAGGGAACA
