# Supplementary figures and images for: FLA1, Enhancing GA3 Contents in Flag Leaf Lamina Joint, Increases Flag Leaf Angle to Improve Outcross Rate and Hybrid Rice Seed Production
Source: Plants (Basel). 2026 Jan 31;15(3):446. doi: 10.3390/plants15030446 (PMC12899354; doi:10.3390/plants15030446)

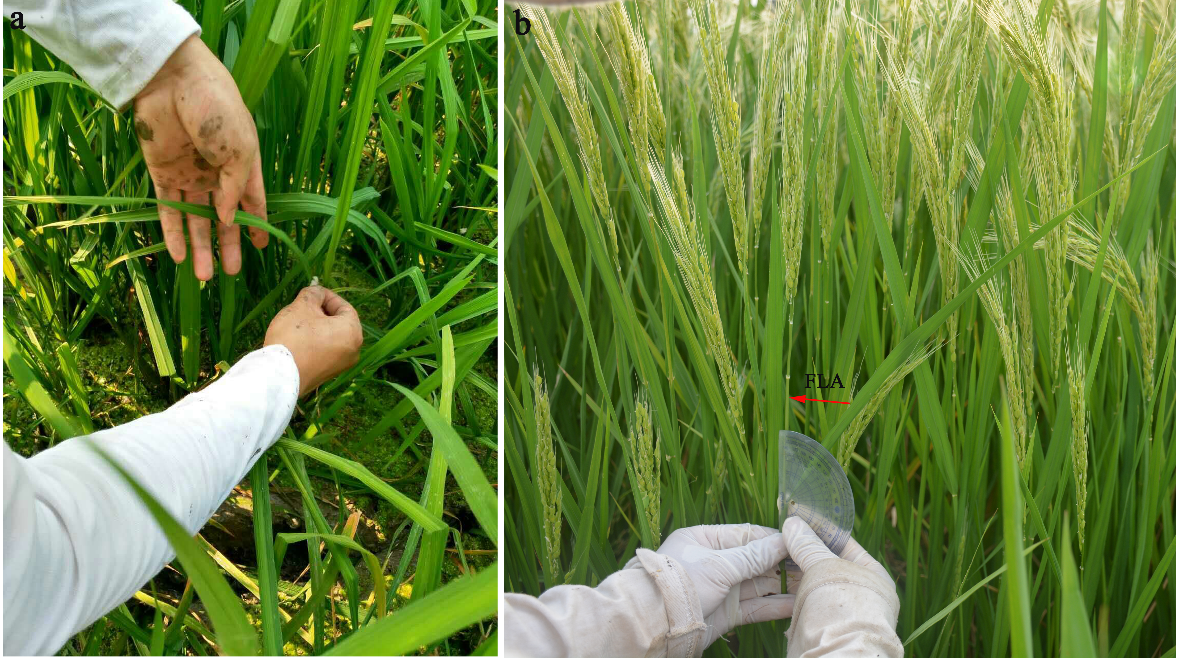

Supplement: Supplementary file 1 [file plants-15-00446-s001.zip › Supplementary Figure S1.tif]

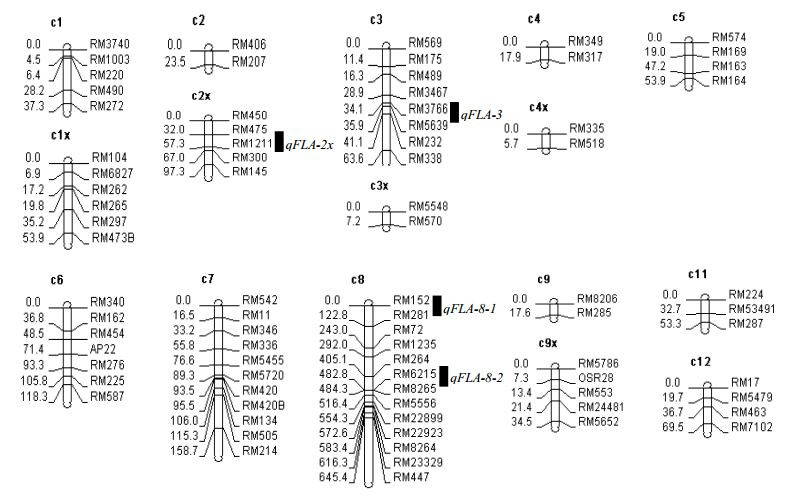

Supplement: Supplementary file 1 [file plants-15-00446-s001.zip › Supplementary Figure S2.jpg]

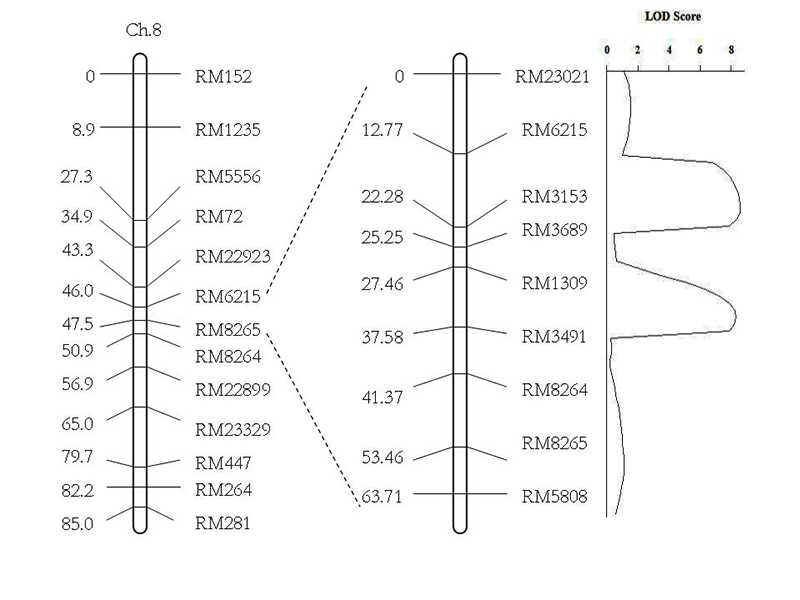

Supplement: Supplementary file 1 [file plants-15-00446-s001.zip › Supplementary Figure S3.tif]

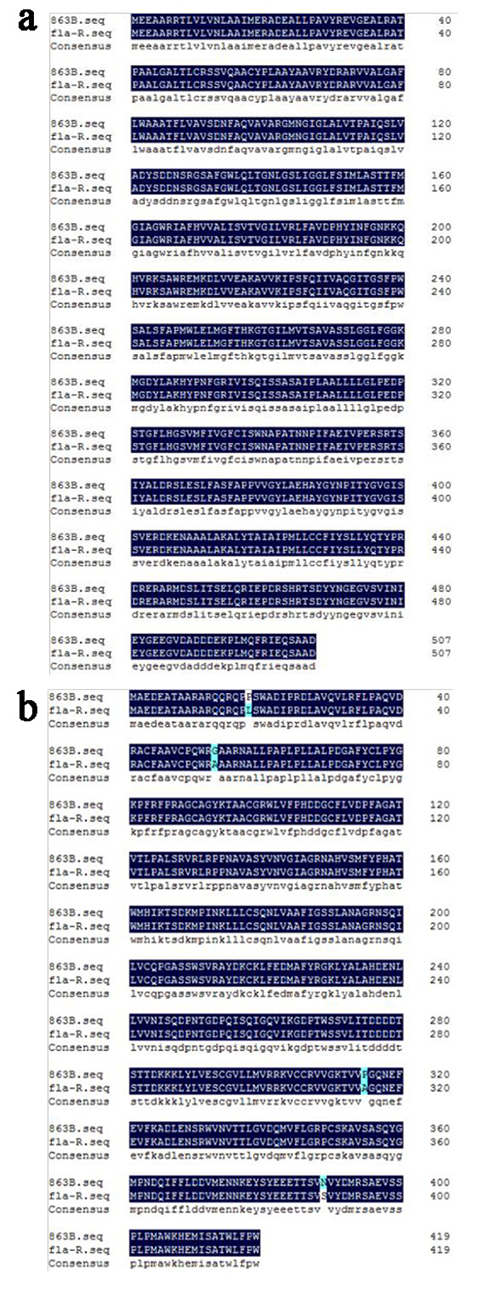

Supplement: Supplementary file 1 [file plants-15-00446-s001.zip › Supplementary Figure S5.tif]

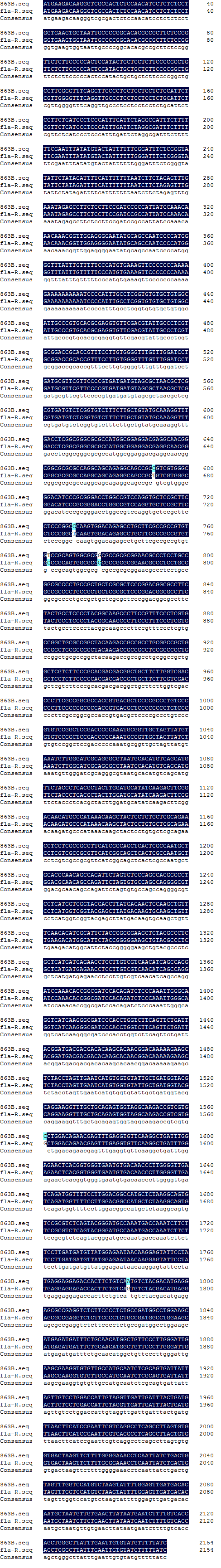

Supplement: Supplementary file 1 [file plants-15-00446-s001.zip › Supplementary Figure S6.tif]
